# Antibacterial Activity for Synthesized Coumarin Derivatives and a Coumarin Component of Lime Peel (*Citrus aurantifolia*)

**DOI:** 10.3390/bioengineering11080752

**Published:** 2024-07-24

**Authors:** Sumi Hwang

**Affiliations:** Department of Biomedical Laboratory Science, College of Health and Medical Science, Sangji University, Won-ju 26339, Republic of Korea; zzz0722@hanmail.net; Tel.: +82-10-4200-6413

**Keywords:** synthesis coumarin derivatives, coumarin component lime peel, antibacterial activity

## Abstract

In this study, we investigated the antibacterial activity of the coumarin component isolated from lime peel and coumarin derivatives synthesized using various techniques against eight types of food-poisoning bacteria. The minimum inhibitory concentration (MIC) for the **3b** [5,7-dihydroxy-4-trifluoromethylcoumarin] derivative was measured as 1.5 mM in *Bacillus cereus, Micrococcus luteus, Listeria monocytogenes,* and *Staphylococcus aureus* subsp. *aureus*; that for the **3c** [7-hydroxy-4-trifluoromethylcoumarin] derivative was 1.7 mM in *Enterococcus facium*; and that for the **3n** [dicoumarol] derivative was 1.2 mM in *L. monocytogenes*. These results confirmed that coumarin derivatives with CF_3_ and OH substituents had enhanced antibacterial activity.

## 1. Introduction

Coumarin is a component involved in plant defense reactions, is a substance that is present in plants, and exhibits various physiological activities. Since ancient times, plant resources such as this have been used to prevent or treat diseases, and in modern times, they are being used in various ways in terms of natural resource use and development.

Accordingly, the coumarin series, which is extracted from the fragrant components of various plants, exhibits various physiological activities, such as antioxidant [1,2], anti-inflammatory [1,2,3,4,5,6,7,8,9,10,11], antibacterial [1,12,13,14,15,16,17,18,19,20], antifungal [21,22,23,24,25], cytotoxic [26,27,28,29,30,31], anticancer [15,32,33,34], anticoagulant [35,36], antimutagenic [37], and photodynamic properties [38,39]. 

Among fragrant fruits, lime peel, which is inexpensive and convenient to purchase, was used as the material for this study. The activity of coumarin derivatives damages cell membranes and shows high activity in Gram-positive and -negative bacteria, but it has been reported to have particularly strong antibacterial activity against Gram-negative bacteria [40,41,42]. In measuring the anti-inflammatory inhibitory activity, it was reported that 6-geranyloxycoumarin had inhibitory effects of 68.9 and 72.6% on the production of interleukin (IL)-6 at 1 and 10 µM, respectively [3]. In addition, as a result of examining the anti-inflammatory effect of geranyloxycoumarin derivatives, natural products, such as 7-geranyloxycoumarin (1 µM/cm^2^) and 8-acetoxt geranyloxycoumarin (1 µM/cm^2^), reduced the edema rate by about 50% [4].

However, detailed studies on the structure–activity relationship (SAR) and new derivatives for these results are lacking. Accordingly, natural coumarin and geranyloxycoumarin were extracted from lime peel (*Citrus aurantifolia*) containing coumarin, separated, and purified, and their structures were analyzed using GC-MS, IR, ^1^H-NMR, ^13^C-NMR, and ^19^F-NMR. We intended to separate the substances and produce coumarin derivatives and geranyloxycoumarin derivatives through synthesis and semi-synthesis.

In this study, the antibacterial activities of coumarin derivatives, geranyloxycoumarin derivatives, and lime peel were confirmed, and it is expected that these results can be used as basic data for antibacterial research on food-poisoning bacteria.

## 2. Materials and Methods

### 2.1. Lime Peel

For the lime peel (*Citrus aurantifolia*) used in this study, 10 kg of raw lime imported from Mexico was obtained and only the peel was collected. This peel was stored and dried in a dryer at 50 °C to minimize changes and then further dried at room temperature. 

### 2.2. Coumarin Derivatives

Hydroxycoumarin, which is a starting material for the synthesis of geranyloxy coumarin derivatives, is manufactured by TCI and the Alfa Aesar company with 97%–98% purity, and 7-hydroxycoumarin (3d), 3-chloro-7-hydroxy-4-methylcoumarin (3f), 6-chloro-7-hydroxy-4-methylcoumarin (3g), 3-phenylumbelliferone (3h), 6-hydroxycoumarin (3i), 6-hydroxy-4-methylcoumarin (3j), 4-methylesculetin (3k), 3-hydroxycoumarin (3l), and 4-hydroxycoumarin (3o) were used. The established methods of synthesis were used to obtain 7-hydroxy-4-trifluoromethyl coumarin (3c), 4-methyl-7-hydroxycoumarin (3e), and dicoumarol (3n). The alkenyl chain used in the synthesis of geranyloxycoumarin derivatives was 95% geranyl bromide (Aldrich). For the base, sodium hydroxide (NaOH), triethylamine (Et_3_N), potassium carbonate (K_2_CO_3_), cesium carbonate (Cs_2_CO_3_), and silver carbonate (Ag_2_CO_3_) were used, whereas the solvents used were acetonitrile, ethyl acetate (EtOAc), dichloromethane, *n*-hexane, acetone, and ethanol (EtOH).

A nuclear magnetic resonance spectrometer (NMR spectrometer; Bruker AVANCE ⅢTM 400 MHz, BRUKER, Germany) was used for analysis. CDCl_3_ and DMSO_d6_ containing tetramethylsilane (TMS), which is an internal standard, were used as analytical solvents. Infrared spectroscopy was performed on an Fourier transform Infrared spectrophotometer (FT-IR spectrophotometer; Jasco FT/IR-4200, JASCO, Tokyo, Japan), and KBr pellets were prepared to confirm the functional groups in the compound.

In addition, the melting point was measured without calibrating the temperature. A thermometer was mounted under a paraffin oil container, and the open glass capillary method was used.

### 2.3. Sample Preparation

#### 2.3.1. Lime Peel Extraction

First, 2 kg of dried lime peel was used in the extraction with 10 L of EtOH at room temperature. The extraction was performed twice, and the extract was vacuum concentrated using a rotary evaporator at 40 °C. Then, 235.8 g of the resulting EtOH extract was mixed with 4 L of *n*-hexane and then vortexed at 500 rpm and 50–60 °C for 6 h. The *n*-hexane layer was isolated, and 34.63 g of concentrate was obtained. A total of 4 L of ethyl acetate was added to the concentrate from which ethanol was completely removed, and after stirring at 50 °C for 6 h, the dissolved layer was separated and concentrated under a 40 °C water bath with the vacuum rotary evaporator, obtaining 15.36 g of concentrate. The remaining residue undissolved in ethyl acetate was then stored in cold storage at 2–4 °C for the purpose of the next study.

#### 2.3.2. Isolation of Lime Peel Components from *n*-Hexane Layer

Silica gels were placed in the column tube, to which 10 g of lime concentrate from the *n*-hexane layer was added after dilution with 30 mL of *n*-hexane. Then, 50 mL of fresh hexane was added (three times), and the column tube was filled with sea sand up to approximately 3 cm. The eluate was produced in the following order: (i) *n*-hexane/CH_2_Cl_2_ = 3:1, *v*/*v*, 2 L; (ii) *n*-hexane/CH_2_Cl_2_ = 2:1, *v*/*v*, 2 L; and (iii) *n*-hexane/CH_2_Cl_2_ = 1:1, *v*/*v*, 2 L, CH_2_Cl_2_ stock 2 L. This was performed to isolate the following components.

#### 2.3.3. Synthesis of Coumarin Derivatives

General coumarin synthesis can be achieved by the Perkin [43,44], Pechmann [45], and Knoevenagel [46,47] reactions. In this study, the following coumarin derivatives with the CF_3_ functional group were synthesized using the Pechmann reaction method (Table 1).

Our first goal was to obtain *O*-alkylated geranyloxycoumarin derivatives through the reaction between hydroxycoumarin and geranyl bromide, and between K_2_CO_3_ and acetone, reacted at room temperature for 5 h to obtain a yield of 35% (Table 2, entry 2). As a result of the optimized conditions, 7-hydroxycoumarin (**3d**) and geranyl bromide (**4**) under Cs_2_CO_3_ and CH_3_CN were reacted for 3 h at room temperature to obtain **5d** in a 93% yield (Table 2, entry 6).

**Figure 1:**
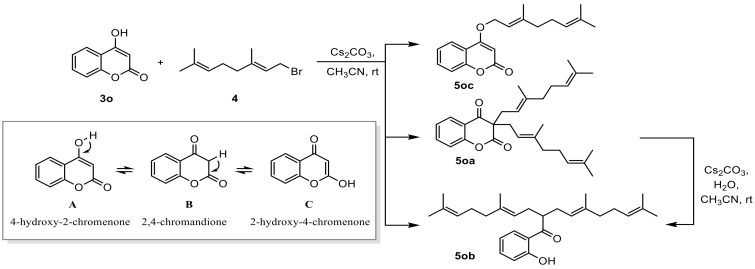
Reactivity of 4-hydroxycoumarin.

Next, we obtained compounds **5oa**, **5ob,** and **5oc** as a result of reacting 4-hydroxycoumarin with geranyl bromide as an optimization condition to obtain *O*-alkylated 4-geranyloxycoumarin. During the formation of these compounds, *O*-alkylated 4-geranyloxycoumarin **5oc** was obtained by reacting A-form coumarin with geranyl bromide by tautomeric ketoenol forms by base. The yield of *O*-alkylated 4-geranyloxycoumarin that was obtained was 18%. On the other hand, C-alkylated coumarin **5oa** was reacted with *2* equivalents of geranyl bromide (**4**) and keto form B to obtain an 11% yield. In addition, compound **5ob** was obtained in a 35% yield by hydrolysis and decarboxylation from **5oa**. We also confirmed that **5ob** was obtained by adding **5oa** to acetonitrile, to which water was added with stirring at rt for 9 h. Using these results, 4-hydroxycoumarin and cinnamyl alcohol were reacted with water and a palladium catalyst to obtain C-alkylated coumarin and diallylated products, which were then hydrolyzed and decarboxylated [48] (Figure 1).

In this result, the reaction of the different types of hydroxy coumarin and geranyl bromide under the given conditions produced various novel coumarin derivatives (Table 3).

### 2.4. Experimental Procedures

#### 2.4.1. Compounds Separated by Lime Peel

The substances isolated from lime peel are as follows. Confirmation of the structure of this material is provided in the Supplementary Materials (Supplementary Table S1).

*(E)-5-((3,7-Dimethylocta-2,6-dien-1-yl)oxy)-7-methoxy-2H-chromen-2-one* ***(5m)***

*5,7-Dimethoxy-2H-chromen-2-one* ***(5m-1)***

(*E)-4-((3,7-dimethylocta-2,6-dien-1-yl)oxy)-7H-furo [3,2-g]chromen-7-one **(5m-2)***

*4,9-Dimethoxy-7H-furo [3,2-g]chromen-7-one(or isopimpinellin) **(5m-3)***

#### 2.4.2. Synthesis of Coumarin Derivatives and Geranyloxycoumarin Derivatives

##### Common Synthesis Method of Coumarin Derivatives

Ethyl trifluoromethyl acetoacetate (20.6 mmol) and phenol derivative (18.2 mmol) were added to a 50 mL round-bottom flask, and the mixture was maintained at below 10 °C. The mixture was stirred while adding 10 mL of sulfuric acid slowly for 30 min. Next, the mixture was stirred at room temperature for 18~26 h and then slowly poured into a beaker containing 80.0 g of iced water, whose temperature was maintained at ≤10 °C. The resulting precipitate was filtered, washed with 10 mL of cold water (four times), and dried in air. The crude solid was purified using column chromatography with CH_2_Cl_2_/*n*-hexane (5:1, *v*/*v*) as the eluent. Confirmation of the structure of this material is provided in the Supplementary Materials (Supplementary Table S2).

*7,8-Dihydroxy-4-trifluoromethylcoumarin **(3a)***

*5,7-Dihydroxy-4-trifluoromethylcoumarin **(3b)***

##### Common Synthesis Method of Geranyloxycoumarin Derivatives

7-hydroxylcoumarin (1.0 mmol), cesium carbonate (1.1 mmol), geranyl bromide (1.2 mmol, 95%), and 30 mL of acetonitrile were added to a 50 mL volumetric flask, and the mixture was stirred at room temperature for 3 h. Subsequently, the solvent was removed using the rotary evaporator. Then, 20 mL of dichloromethane was added to the mixture, which was filtered and then concentrated using the rotary evaporator. Geranyloxycoumarin was purified by silica gel column chromatography using CH_2_Cl_2_/*n*-hexane (1:1, *v*/*v*) as the eluent [46]. Confirmation of the structure of this material is provided in the Supplementary Materials (Supplementary Table S3).

*(E)-7-((3,7-dimethylocta-2,6-dien-1-yl)oxy)-4-(trifluoromethyl)-2H-chromen-2-one **(5c)***

*(E)-7-((3,7-dimethylocta-2,6-dien-1-yl)oxy)-2H-chromen-2-one **(5d**)* [47]

*(E)-7-((3,7-dimethylocta-2,6-dien-1-yl)oxy)-4-methyl-2H-chromen-2-one **(5e)*** [48]

*(E)-3-chloro-7-((3,7-dimethylocta-2,6-dien-1-yl)oxy)-4-methyl-2H-chromen-2-one**(5f**)* [49]

*(E)-6-chloro-7-((3,7-dimethylocta-2,6-dien-1-yl)oxy)-4-methyl-2H-chromen-2-one **(5g)***

*(E)-7-((3,7-dimethylocta-2,6-dien-1-yl)oxy)-3-phenyl-2H-chromen-2-one **(5h**)*

*(E)-6-((3,7-dimethylocta-2,6-dien-1-yl)oxy)-4-methyl-2H-chromen-2-one **(5j**)* [47,48]

*6-(((E)-3,7-dimethylocta-2,6-dien-1-yl)oxy)-7-(((Z)-3,7-dimethylocta-2,6-dien-1-yl)oxy)-4-*

*methyl-2H-chrom* *en-2-one **(5k**)*

*(E)-3-((3,7-dimethylocta-2,6-dien-1-yl)oxy)-2H-chromen-2-one **(5l**)* [47]

*3,3-Bis((E)-3,7-dimethylocta-2,6-dien-1-yl)chromane-2,4-dione**(5oa**)* [50]

*(E)-2-((E)-3,7-dimethylocta-2,6-dien-1-yl)-1-(2-hydroxyphenyl)-5,9-dimethyldeca-4,8-*

*dien-1-one**(5o*** ***b)***

*(E)-4-(3,7-dimethylocta-2,6-dienyloxy)-2H-chromen-2-one **(5oc)***

##### Synthesis Method of (E)-2-((E)-3,7-Dimethylocta-2,6-dien-1-yl)-1-(2-hydroxyphenyl)-5,9-dimethyldeca-4,8-dien-1-one (**5ob**)

Distilled water (2.6 mmol) was added to a stirred mixture of **5oa** (2.3 mmol) and powdered cesium carbonate (2.5 mmol) in acetonitrile (30 mL), and the stirring was continued at rt for 9 h. The reaction progress was monitored using TLC. The resulting mixture was filtered, and the filtrate was concentrated. The crude product was fractionated on a silica gel column using *n*-hexane/CH_2_Cl_2_ (3:1, *v*/*v*) to give the product 5ob as a colorless liquid.

### 2.5. Evaluating the Antibacterial Activity

#### 2.5.1. Test Strain

The following microorganisms were used to evaluate the antibacterial activity: five strains of Gram-positive bacteria (*B. cereus, M. luteus, E. faecium, L. monocytogenes, S. aureus* subsp. *aureus*) and three strains of Gram-negative bacteria (*Salmonella enteritidis, Shigella boydii, E. coli*) as the common pathogens. The strains used in this study were obtained from the Korean Collection for Type Cultures (KTCT) and Korean Culture Center of Microorganisms (KCCM).

#### 2.5.2. Microbial Culture

The obtained pathogenic microorganisms were cultured under standard microbial culture conditions, and after passaging in each medium, a preculture was performed at 37 °C and 150 rpm for 12 h. Each microbial strain was then cultured up to 6.4 × 10⁶ CFU/mL for subsequent analyses. The composition of each medium for evaluating the antimicrobial activity is listed in Table 4.

#### 2.5.3. Measurement of Antibacterial Activities

##### Screening Test for Coumarin Derivatives

The final concentration of coumarin was set to 10 mg/mL, and the inhibitory activities for *B. cereus*, *M. luteus*, *L. monocytogenes*, and *E. faecium* were evaluated to test the activity of the indicator strains according to the concentration of coumarin derivatives. For each medium and optimal temperature, the indicator strains were cultured in a shaking incubator for 12 h. Thereafter, a 1% culture solution of each indicator strain was added to 0.8% soft agar, and 20 mL of agar was solidified in a Petri dish. The test plate for antibacterial activity had 8 mm wells, and 50 μL of a sample was loaded into each well and cultured at the optimal temperature for 12 h. The indicator strains showing transparent circular colonies were selected and their diameters were measured. The results are expressed as the measured values of the screening test (-: no activity; +: inhibition zone >5–10 mm; ++: inhibition zone >11–15 mm; +++: inhibition zone >16–20 mm; and ++++: inhibition zone >20 mm).

DMSO was used as the negative control to investigate the effect of inhibiting proliferation on the solvent.

##### Minimum Inhibitory Concentration (MIC)

The final concentration of coumarin was set to 0.09, 0.19, 0.39, 0.78, 1.56, 3.12, 6.25, 12.5, 25, 50, and 100 mg/mL, and the inhibitory activities for *M. luteus, L. monocytogenes, E. faecium*, and *B. cereus* were evaluated to determine the MIC of the indicator strains according to the concentration of coumarin derivatives.

For each medium and optimal temperature, the indicator strains were cultured in a shaking incubator for 12 h. To 0.8% soft agar, 1% culture solution of each indicator strain was added, and 20 mL of agar was solidified in a Petri dish. The test plate for antimicrobial activity had 8 mm wells, and 50 μL of a sample was loaded into each well and cultured at the optimal temperature for 12 h. The lowest concentration at which the transparent circular colonies appeared was considered as the MIC. The results are expressed by converting the concentration in mg/mL into a molar concentration.

DMSO was used as the negative control to investigate the effect of inhibiting proliferation on the solvent.

## 3. Results and Discussion

### 3.1. Synthesis

In the present study, various coumarin derivatives with a geranyloxy group were synthesized and their bioactivities were compared to investigate the diversity of the potential pharmacological effects of 7-methoxy-5-geranyloxycoumarin and bergamottin isolated from lime.

In this study, the reaction between 7-hydroxycoumarin (**3d**) and geranyl bromide (**4**) was studied in the synthesis of the geranyloxycoumarin derivative (**5d**) using both weak and strong base additives, such as K_2_CO_3_, Cs_2_CO_3_, and Ag_2_CO_3_, respectively. From these results, we obtained 95% yields for the O-alkylated geranyloxycoumarin derivatives (**5a~5l**) by reacting various hydroxycoumarins (**3a~3l**) with geranyl bromide (**4**) under optimal reaction conditions. However, the reaction of 4-hydroxycoumarin (**3o**) and geranyl bromide under mild optimized reaction conditions obtained **5oa, 5ob**, and **5oc**. It was reported that the reason for the formation of compounds such as **5oa, 5ob**, and **5oc** is that various coumarin derivatives are produced due to the keto enol tautomerism of 4-hydroxycoumarin (**3o**) [51].

### 3.2. Measurement of Antibacterial Activities of Coumarin Derivatives

#### 3.2.1. Screening Test for Coumarin Derivatives, Geranyloxycoumarin Derivatives, and Lime Peel

The antibacterial activities for coumarin derivatives, geranyloxycoumarin derivatives, and lime peel were assessed against eight species of food-poisoning bacteria.

The screening test shown in Table 5 and Table 6 indicates that the coumarin derivatives showed good antibacterial activity. From the culture results, *B. cereus* showed the highest antibacterial activity among the indicator bacteria (++++: inhibition zone >20 mm) when using 3a, 3b, 3c, 3d, 3e, 3j, and 3n. Among them, 3a affected *B. cereus*, *M. luteus,* and *S. aureus* subsp. *aureus*; 3b affected *B. cereus*, *M. luteu, L. monocytogenes,* and *S. aureus* subsp. *aureus*; 3n affected *B. cereus* and *S. aureus* subsp. *aureus*; and 3c, 3d, 3e, and 3j showed an antibacterial activity of ++++: inhibition zone >20 mm in *B. cereus*. As a result, the antibacterial activity of the coumarin derivatives (3a, 3b, 3c) containing CF_3_ was confirmed.

DMSO was used as the negative control to investigate the effect of inhibiting proliferation on the solvent.

#### 3.2.2. Minimum Inhibitory Concentration (MIC) of Coumarin Derivatives

To measure the MIC of the coumarin derivative with antibacterial activity, the final concentration of coumarin was adjusted to 0.09, 0.19, 0.39, 0.78, 1.56, 3.12, 6.25, 12.5, 25, 50, and 100 mg/mL, and then the inhibitory activity was measured. The results are expressed by converting the concentration in mg/mL into a molar concentration.

According to Table 7, the MIC of **3b** was 1.5 mM in *B. cereus*, *M. luteus*, *L. monocytogenes*, and *S. aureus* subsp. *aureus*. The MIC of **3c** was 1.7 mM in *E. faecium*, and that of **3n** was 1.2 mM in *L. monocytogenes*.

As a result of the MIC measurement, the antibacterial activity was confirmed in the 3b and 3c derivatives. These results confirmed that coumarin derivatives with CF_3_ and OH substituents had antibacterial activity against food-poisoning bacteria.

## 4. Conclusions

In this study, 5,7-dimethoxy coumarin, 5-geranyloxy-7-methoxy coumarin, isopimpinellin, and bergamottin were isolated from lime peel. In addition, coumarin derivatives and geranyloxycoumarin, which are expected to have various biological activities, were synthesized in excellent yields using optimized Cs_2_CO_3_, acetonitrile, and room temperature (20 ± 5 °C).

The antibacterial screening of coumarin derivatives, geranyloxycoumarin derivatives, and the coumarin component of lime peel was initiated on eight species of food-poisoning bacteria, and the MIC of the coumarin derivative with good activity was measured. As a result of the MIC measurement, antibacterial activity was confirmed in the 3b and 3c derivatives. These results confirm that coumarin derivatives with CF_3_ and OH substituents had antibacterial activity against food-poisoning bacteria.

Antibacterial activity against *Enterococcus faecalis* has been reported only for 1,2,3-triazole-coumarin derivatives [16]. However, the results of the antibacterial screening conducted in this study confirm that coumarin derivatives exhibited antibacterial effects on *B. cereus, S. aureus* subsp. *aureus*, *M. luteus*, *S. enteritidis*, and *S. boydii*.

In addition, the water-soluble coumarin quaternary ammonium chloride was synthesized against Gram-negative *E. coli* and Gram-positive *B. subtilis* bacteria, and it was reported that no antibacterial activity was observed [52]. However, the antibacterial screening results of compound 3a showed that it exhibited activity toward *E. coli*. As a result of this experiment, the MIC of the 3b derivative was measured at a concentration of 2.9 mM in *E. coli*.

The results obtained herein imply that **3b** and **3c** derivatives, which contain CF_3_ substituents, can be utilized as natural antibacterial substances, as their antibacterial activity was confirmed. We believe that the large-scale synthesis of coumarin derivatives with antibacterial activity will be possible if the various derivative synthetic compounds we have proposed are utilized in further research.

Accordingly, in this study, we attempted to identify a method with a high recovery rate using a coumarin synthesis method and then confirmed the antibacterial activity using a coumarin derivative. These results are expected to provide useful information on antibacterial activity using coumarin derivatives that are water soluble and contain fluorine (F). In the future, it is expected that useful conditions to achieve a better antibacterial activity can be found by synthesizing additional derivatives utilizing structure–activity relationships.

## Figures and Tables

**Table 1:** Synthesis of hydroxycoumarin.

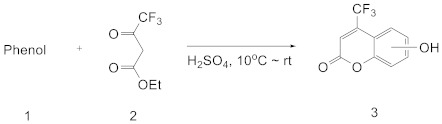			
No.	Phenol (1)	Product (3)	Yield (%)
1	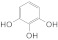 **1a**	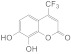 **3a**	72
2	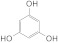 **1b**	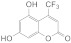 **3b**	66
3	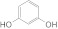 **1c**	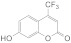 **3c**	78

**Table 2:** Optimization reaction conditions for **5d**.

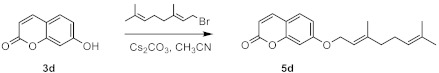		
Entry	Conditions	Yield (%)
1	TEA, acetone, rt, 12 h	Degradation
2	K_2_CO_3_, acetone, rt, 5 h	35
3	K_2_CO_3_, acetone, rt, 26 h	62
4	K_2_CO_3_, acetone, reflux, 1 h	73
5	K_2_CO_3_, CH_3_CN, reflux, 1 h	74
6	Cs_2_CO_3_, CH_3_CN, rt, 3 h	93
7	Cs_2_CO_3_, CH_3_CN, reflux, 30 min	87
8	Ag_2_CO_3_, CH_3_CN, rt, 3 h	85

**Table 3:** Synthesized geranyloxycoumarin derivative.

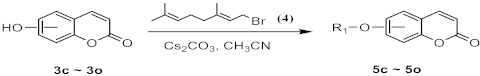							
No.	Coumarin	No.	Product	No.	Coumarin	No.	Product
3c	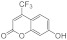	**5c**	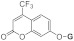	3k	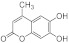	**5k**	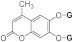
3d	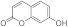	**5d**	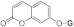	3l	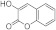	**5l**	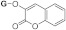
3e	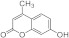	**5e**	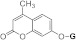	3m	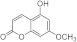	**5m**	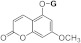
3f	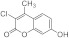	**5f**	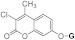	3n	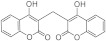	**5od**	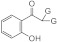
3g	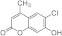	**5g**	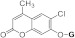	3o	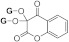	**5oa**	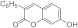
3h	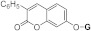	**5h**	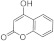			**5oc**	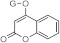
3i	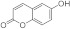	**5i**	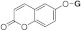			**5od**	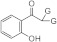
3j	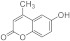	**5j**	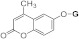				
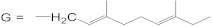							

**Table 4:** Growth conditions of pathogenic bacteria.

Organism	Condition	°C
*Bacillus cereus* KCCM 11204	ENB (yeast extract 0.25% + Brain Heart Infusion broth 1.25% + Nutrient broth 0.55%)	30
*Micrococcus luteus* IAM 1056	ENB (yeast extract 0.25% + Brain Heart Infusion broth 1.25% + Nutrient broth 0.55%)	30
*Enterococcus faecium* KCCM 12118	BHI (Brain Heart Infusion broth)	37
*Listeria monocytogenes* KCCM 40307	BHI (Brain Heart Infusion broth)	37
*Salmonella enteritidis* KCCM 12021	Nutrient broth	37
*Shigella boydii* KCCM 41649	Nutrient broth	37
*Escherichia coli* KCCM 11835	LB broth	37
*Staphylococcus aureus* subsp. *aureus* KCCM 40050	LB broth	37

**Table 5:** Screening test of coumarin derivatives in 10 mg/mL.

Test Organisms				Coumarin Derivatives												
	DMSO	3a	3b	3c	3d	3e	3f	3g	3h	3i	3j	3k	3l	3n	3o	3p
***B. cereus*** **KCCM 11204**	**-**	**++++**	**++++**	**++++**	**++++**	**++++**	**+++**	**++**	**++**	**++**	**++++**	**-**	**+++**	**++++**	**++**	**++**
***M. luteus*** **IAM 1056**	**-**	**++++**	**++++**	**+++**	**-**	**+**	**+**	**-**	**+**	**-**	**++**	**++**	**+**	**+++**	**-**	**++**
***L. monocytogenes*** **KCCM 40307**	**-**	**+++**	**++++**	**++**	**-**	**+**	**+**	**+**	**+**	**+**	**+**	**+**	**++**	**++**	**++**	**+**
***E. faecium*** **KCCM 12118**	**-**	**+++**	**+++**	**++**	**-**	**++**	**+**	**-**	**+**	**+**	**+**	**+**	**++**	**++**	**+**	**+**
***S. enteritidis*** **KCCM 12021**	**-**	**++**	**+**	**++**	**-**	**++**	**-**	**-**	**++**	**+**	**++**	**++**	**+++**	**-**	**-**	**++**
***S. boydii*** **KCCM 41649**	**-**	**++**	**+**	**++**	**-**	**+++**	**-**	**-**	**++**	**-**	**++**	**++**	**+++**	**-**	**-**	**+++**
***E. coli*** **KCCM 11835**	**-**	**+++**	**+**	**+**	**-**	**++**	**-**	**-**	**++**	**-**	**++**	**++**	**+**	**-**	**+**	**++**
***S. aureus*** **subsp. *aureus* KCCM 40050**	**-**	**++++**	**++++**	**+++**	**-**	**++**	**+**	**-**	**++**	**-**	**++**	**-**	**++**	**++++**	**-**	**++**

The criteria for the area of antibacterial activity inhibition are as follows. -: no activity; +: inhibition zone >5–10 mm; ++: inhibition zone >11–15 mm; +++: inhibition zone >16–20 mm; ++++: inhibition zone >20 mm.

**Table 6:** Screening test of geranyloxycoumarin derivatives and coumarin component of lime peel in 10 mg/mL.

Test Organisms		Geranyloxycoumarin Derivatives										Lime Peel			
	DMSO	5c	5d	5e	5f	5g	5h	5i	5j	5k	5l	5m	5m-1	5m-2	5m-3
***B. cereus* KCCM 11204**	**-**	**+**	**++**	**+**	**++**	**+**	**+**	**+**	**+**	**-**	**+**	**+**	**+**	**+**	**+**
***M. luteus* IAM 1056**	**-**	**+**	**+**	**+**	**+**	**+**	**+**	**+**	**+**	**+**	**+**	**+**	**+**	**+**	**+**
***L. monocytogenes* KCCM 40307**	**-**	**+**	**+**	**+**	**+**	**+**	**+**	**+**	**+**	**+**	**+**	**+**	**+**	**+**	**+**
***E. faecium* KCCM 12118**	**-**	**+**	**+**	**+**	**+**	**++**	**+**	**+**	**+**	**+**	**+**	**+**	**+**	**+**	**+**
***S. enteritidis* KCCM 12021**	**-**	**-**	**-**	**-**	**-**	**-**	**-**	**-**	**-**	**-**	**-**	**-**	**-**	**-**	**-**
***S. boydii* KCCM 41649**	**-**	**+**	**-**	**-**	**-**	**-**	**-**	**-**	**-**	**-**	**-**	**-**	**-**	**-**	**+**
***E. coli* KCCM 11835**	**-**	**+**	**+**	**+**	**+**	**-**	**+**	**-**	**+**	**+**	**+**	**+**	**+**	**+**	**+**
***S. aureus* subsp. *aureus* KCCM 40050**	**-**	**-**	**-**	**-**	**-**	**-**	**-**	**-**	**-**	**-**	**-**	**-**	**-**	**-**	**-**

The criteria for the area of antibacterial activity inhibition are as follows. -: no activity; +: inhibition zone >5–10 mm; ++: inhibition zone >11–15 mm.

**Table 7:** Minimum inhibitory concentration (MIC) of coumarin derivatives (mM).

	DMSO	*B. cereus* KCCM 11204	*M. luteus* IAM 1056	*L. monocytogenes* KCCM 40307	*E. faecium* KCCM 12118	*S. enteritidis* KCCM 12021	*S. boydii* KCCM 41649	*E. coli* KCCM 1835	*S. aureus* subsp. *aureus* KCCM 40050
**3a**	-	23	-	-	8.1	30	46	-	-
**3b**	-	1.5	1.5	1.5	2.9	-	-	2.9	1.5
**3c**	-	3.3	12.7	3.3	1.7	6.7	-	-	3.3
**3d**	-	44	30	7.8	4.2	7.8	3.6	7.8	61
**3e**	-	23	23	3.6	7.9	7.9	7.9	3.3	50
**3j**	-	42	28	19	14	19	3.3	3.3	7.9
**3k**	-	50	50	38	-	50	-	-	10
**3n**	-	27	3.7	1.2	3.7	27	14	-	2.9
**3o**	-	46	15.1	7.6	7.8	3.3	7.8	7.8	7.8

-: no activity, Statistical analysis was performed using the ANOVA 26 VERSION SPSS program. The mean and standard deviation were calculated for each item, and significance was verified using two-way ANOVA (*p* < 0.05). Values presented are means ± standard errors from three independent experiments.

## Data Availability

Data are available upon appropriate request to the author.

## Supplementary Materials

The following supporting information can be downloaded from https://www.mdpi.com/article/10.3390/bioengineering11080752/s1. Table S1: Compounds separated by lime. Table S2: Common synthesis method of coumarin derivatives. Table S3: Common synthesis method of geranyloxy coumarin derivative.

## References

[1] Alshibl H.M., Al-Abdullah E.S., Haiba M.E., Alkahtani H.M., Awad G.E.A., Mahmoud A.H., Ibrahim B.M.M., Bari A., Villinger A. (2020). Synthesis and Evaluation of New Coumarin Derivatives as Antioxidant, Antimicrobial, and Anti-Inflammatory Agents. Molecules.

[2] Tanimoto A., Witaicenis A., Caruso Í., Piva H.M.R., Araujo G.C., Moraes F.R., Fossey M.C., Cornélio M.L., Souza F.P., Stasi L.C.D. (2020). 4-Methylesculetin, a natural coumarin with intestinal anti-inflammatory activity, elicits a glutathione antioxidant response by different mechanisms. J. Chem. Biol. Interact..

[3] Kim D.S. (2012). A Study on the Efficacy of the Coumarine Derivatives with Anti-Inflammatory Activity in the Trifoliate Orange Extract. J. Korean Oil Chem. Soc..

[4] Curini M., Epifano F., Maltese F., Marcotullio M.C., Tubaro A., Altinier G., Gonzales S.P., Rodriguez J.C. (2004). Synthesis and anti-inflammatory activity of natural and semisynthetic geranyloxycoumarins. Bioorganic Med. Chem..

[5] Stasi L.C.D. (2021). Coumarin Derivatives in Inflammatory Bowel Disease. Molecules.

[6] Okuyama S., Morita M., Kaji M., Amakura Y., Yoshimura M., Shimamoto K., Ookido Y., Nakajima M., Furukawa Y. (2015). Auraptene Acts as an Anti-Inflammatory Agent in the Mouse Brain. Molecules.

[7] Anh H.L.T., Kim D.C., Ko W.M., Ha T.M., Nhiem N.X., Yen P.H., Tai B.H., Truong L.H., Long V.N., Gioi T. (2017). Antiinflammatory coumarins from Paramignya trimera. J. Pharm. Biol..

[8] Kirsch G., Abdelwahab A.B., Chaimbault P. (2016). Natural and Synthetic Coumarins with Effects on Inflammation. Molecules.

[9] Jang S.L., Kim H.J., Hwang K.M., Pae H.O., Yun Y.G., Chung H.T., Kim Y.C. (2002). Anti-Inflammatory Effect of Ethanol Extract of Angelica uchiyamana in Activated Murine RAW 264.7 macrophages. J. Korean Med. Soc. Herb. Formula Study.

[10] Khatib A., Kim M.Y., Chung S.K. (2005). Anti-inflammatory Activities of *Cinanamomum burmanni* BI. J. Food Sci. Biotechnol..

[11] Grovera J., Jachak S.M. (2015). Coumarins as privileged scaffold for anti-inflammatory drug development. J. RSC Adv..

[12] Qin H.L., Zhang Z.W., Ravindar L., Rakesh K.P. (2020). Antibacterial activities with the structure-activity relationship of coumarin derivatives. Eur. J. Med. Chem..

[13] Arshad A., Osman H., Bagley M.C., Lam C.K., Mohamad S., Zahariluddin A.S.M. (2011). Synthesis and antimicrobial properties of some new thiazolyl coumarin derivatives. Eur. J. Med. Chem..

[14] Lipeeva A.V., Zakharov D.O., Burova L.G., Frolova T.S., Baev D.S., Shirokikh I.V., Evstropov A.N., Sinitsyna O.I., Tolsikova T.G., Shults E.E. (2019). Design, Synthesis and Antibacterial Activity of Coumarin-1,2,3-triazole Hybrids Obtained from Natural Furocoumarin Peucedanin. Molecules.

[15] Stojković D.L., Jevtić V.V., Vuković N., Vukić M., Čanović P., Zarić M.M., Mišić M.M., Radovanović D.M., Baskić D., Trifunović S.R. (2018). Synthesis, characterization, antimicrobial and antitumor reactivity of new palladium (II) complexes with methionine and tryptophane coumarin derivatives. J. Mol. Struct..

[16] López-Rojas P., Janeczko M., Kubiński K., Amesty Á., Masłyk M., Estévez-Braun A. (2018). Synthesis and Antimicrobial Activity of 4-Substituted 1,2,3-Triazole-Coumarin Derivatives. Molecule.

[17] Céspedes C.L., Avila J.G., Martínez A., Serrato B., Calderón-Mugica J.C., Salgado-Garciglia R. (2006). Antifungal and antibacterial activities of Mexican tarragon (*Tagetes lucida*). J. Agric. Food Chem..

[18] Saleem M., Nazir M., Ali M.S., Hussain H., Lee Y.S., Riaz N., Jabbar A. (2010). Antimicrobial natural products: An update on future antibiotic drug candidates. Nat. Prod. Rep..

[19] Yu Y.M. (2015). The Antibacterial Effects and Mechanism of Several Botanical Compounds against *Ralstonia solanacearum*. Master ’s Thesis.

[20] Chita R.S., Jyotirmaya S., Monalisa M., Debananda L., Pratap K.S., Budheswar D. (2021). Coumarin derivatives as promising antibacterial agent(s). Arab. J. Chem..

[21] Guerra F.Q.S., Araújo R.S.A., Sousa J.P., Silva V.A., Pereira F.O., Mendonça-Junior F.J.B., Barbosa-Filho J.M., Pereira J.A., Lima E.O. (2018). A new coumarin derivative, 4-acetatecoumarin, with antifungal activity and association study against *Aspergillus spp*. Braz. J. Microbiol..

[22] Ramírez-Pelayo C., Martínez-Quiñones J., Gil J., Durango D. (2019). Coumarins from the peel of citrus grown in Colombia: Composition, elicitation and antifungal activity. J. Heliyon.

[23] Kurdelas R.R., Lima B., Tapia A., Feresin G.E., Sierra M.G., Rodríguez M.V., Zacchino S., Enriz R.D., Freile M.L. (2010). Antifungal activity of extracts and prenylated coumarins isolated from Baccharis darwinii Hook & Arn. (Asteraceae). Molecules.

[24] De Araújo R.S.A., Guerra F.Q.S., Lima E.D.O., De Simone C.A., Tavares J.F., Scotti L., Scotti M.T., De Aquino T.M., De Moura R.O., Mendonça F.J.B. (2013). Synthesis, structure-activity relationships (SAR) and in silico studies of coumarin derivatives with antifungal activity. Int. J. Mol. Sci..

[25] Guerra F.Q.S., de Araújo R.S.A., de Sousa J.P., de Oliveira Pereira F., Mendonça-Junior F.J.B., Barbosa-Filho J.M., de Oliveira Lima E. (2015). Evaluation of Antifungal Activity and Mode of Action of New Coumarin Derivative, 7-Hydroxy-6-nitro-2H-1-benzopyran-2-one, against Aspergillus spp. Evid. Based Complement. Altern. Med..

[26] Kostova I. (2002). Synthetic and natural coumarins as cytotoxic agents. Curr. Med. Chem. Anticancer. Agents.

[27] Manolov I., Kostova I., Netzeva T., Konstantinov S., Karaivanova M. (2000). Cytotoxic activity of cerium complex with coumarin derivatives Molecular modeling of the ligands. Archiv. Pharm. Med. Chem..

[28] Lake B.G. (1999). Coumarin metabolism, toxicity and carcinogenicity: Relevance for human risk assessment. Food Chem. Toxicol..

[29] Gardelly M., Trimech B., Belkacem M.A., Harbach M., Abdelwahed S., Mosbah A., Bouajila J., Ben Jannet H. (2016). Synthesis of novel diazaphosphinanes coumarin derivatives with promoted cytotoxic and anti-tyrosinase activities. Bioorganic Med. Chem. Lett..

[30] You C.X., Yang K., Wang C.F., Zhang W.J., Wang Y., Han J., Fan L., Du S.S., Geng Z.F., Deng Z.W. (2014). Cytotoxic Compounds Isolated from Murraya tetramera Huang. Molecules.

[31] Baik K.U., Ahn B.Z. (1988). Cytotoxic Activities of some Geranylated Flavones against L1210 Cell. J. Yakhak Hoeji.

[32] Lee J.H., Lee J.H., Kim H.K., Kim E.G., Cho S.H. (2006). Synthesis of Coumarin Analogues and their Antitumor Activity. J. YAKHAK HOEJI.

[33] Prashanth T., VijayAvin B.R., Thirusangu P., Ranganatha V.L., Prabhakar B.T., Narendra J.N., Chandra S., Khanum S.A. (2019). Synthesis of coumarin analogs appended with quinoline and thiazole moiety and their apoptogenic role against murine ascitic carcinoma. J. Biomed. Pharmacother..

[34] Peperidou A., Bua S., Bozdag M., Hadjipavlou-Litina D., Supuran C.T. (2018). Novel 6- and 7-Substituted Coumarins with Inhibitory Action against Lipoxygenase and Tumor-Associated Carbonic Anhydrase IX. Molecules.

[35] Kasperkiewicz K., Ponczek M.B., Owczarek J., Guga P., Budzisz E. (2020). Antagonists of Vitamin K—Popular Coumarin Drugs and New Synthetic and Natural Coumarin Derivatives. Molecules.

[36] Oldenburg J., Seidel H., Pötzsch B., Watzka M. (2008). New insight in therapeutic anticoagulation by Coumarin derivatives. Hamostaseologie.

[37] Marques A.D.S., Lin C.T. (2004). Molecular complexes of IQ and 4-hydroxycoumarin: A mutagen-anti-mutagen system. J.Photochem. Photobiol..

[38] Chool B.Y., Song K.S. (2012). UVB-Shielding Effects of para-Coumaric Acid. Cosmet. Sci. Korea.

[39] Jung O.U., Lee S.S. (2013). Preparation of Dye Sensitized Solar Cell Using Coumarin Dyes Extracted from Plants. Korea Chem. Eng. Res..

[40] Sumi H., Eonjoo R. (2022). Synthesis of Geranyloxycoumarin Derivatives under Mild Conditions Using Cs_2_CO_3_. J. Turk. Chem. Soc. Chem. A.

[41] Iranshahi M., Jabbari A., Orafaie A., Mehri R., Zeraatkar S., Ahmadi T., Alimardani M., Sadeghian H. (2012). Synthesis and SAR studies of o-prenylated coumarins as potent 15-lipoxygenase inhibitors. Eur. J. Med. Chem..

[42] Khomenko T.M., Zarubaev V.V., Orshanskaya I.R., Kadyrova R.A., Sannikova V.A., Korchagina D.V., Volcho K.P., Salakhutdinov N.F. (2017). Anti-influenza activity of monoterpene-containing substituted coumarins. Bioorganic Med. Chem. Lett..

[43] Ghulam R. (2018). A Concise Introduction of Perkin Reaction. Org. Chem. Curr. Res..

[44] He X., Yan Z., Hu X., Zuo Y., Jiang C., Jin L., Shang Y. (2014). FeCl3-Catalyzed Cascade Reaction: An Efficient Approach to Functionalized Coumarin Derivatives. Synth. Commun..

[45] Nirajkumar H.J., Sachin S.S., Nishant K.R., Dnyaneshwar R.S., Ramdas A.P. (2019). Heterogeneously Catalyzed Pechmann Condensation Employing the Tailored Zn0.925Ti0.075O NPs: Synthesis of Coumarin. ACS Omega.

[46] Abraham G.G., David M., Aparicio S., Hidemí A.M., Abraham G.R., Luis F.R., Cuauhtémoc A.S., Cárlos E., Lobato G., Nancy R.C. (2016). Synthesis of 3-carboxylated Coumarins by Knoevenagel Condensation and Exploratory Anti-inflammatory Activity Evaluation by in vivo model. Am. J. Org. Chem..

[47] Vekariya R.H., Patel H.D. (2014). Recent advances in the synthesis of coumarin derivatives via Knoevenagel condensation: A review. Synth. Commun..

[48] Shue Y.J., Shyh-Chyun Y. (2012). Activator-free and one-pot C-allylation by simple palladium catalyst in water. Tetrahedron Lett..

[49] Orhan I.E., Deniz F.S.S., Salmas R.E., Durdagi S., Epifano F., Genovese S., Fiorito S. (2019). Combined molecular modeling and cholinesterase inhibition studies on some natural and semisynthetic O-alkylcoumarin derivatives. Bioorg. Chem..

[50] Venturella P., Bellino A., Luisa M.M. (1982). Synthesis of terpenoid coumarins, an approach to the synthesis of Piloselliodan. Gazz. Chim. Ital..

[51] Cravotto G., Nano G., Palmisano G., Tagliapietra S. (2003). 4-Hydroxycoumarin and Related Systems: Sitoselectivity of the Mitsunobu Reaction with Prenyl Alcohols. Heterocycles.

[52] Karataş M.O., Noma S.S.S., Gürses C., Balcıoğlu S., Ateş B., Alıcı B., Çakır Ü. (2020). Water Soluble Coumarin Quaternary Ammonium Chlorides: Synthesis and Biological Evaluation. Chem. Biodivers..

