# Supplementary material for: Antibacterial Activity for Synthesized Coumarin Derivatives and a Coumarin Component of Lime Peel (Citrus aurantifolia)
Source: Bioengineering (Basel). 2024 Jul 24;11(8):752. doi: 10.3390/bioengineering11080752 (PMC11351996; doi:10.3390/bioengineering11080752)
Supplement: Supplementary file 1 [file bioengineering-11-00752-s001.zip › bioengineering-3078864-supplementary.pdf]

Supplementary Materials

Table S1. Compounds separated by lime.

Compound	Material form	m.p. (°C)	IR (KBr, cm^{-1})	^{1}H-NMR (400 MHz, $CDCl_3$)	^{13}C-NMR (100 MHz, $CDCl_3$)	MS *m/z*	CHN analysis
5m	White powder	86-87	1726 (>C=O), 1606, 1437, 1040	δ 1.61 (s, CH_3), 1.68 (s, CH_3), 1.75 (s, CH_3), 2.07-2.16 (m, 4H, -CH_2CH_2-), 3.85 (s, OCH_3), 4.60 (d, *J* = 7.2 Hz, -CH_2-), 5.05-5.11 (m, 1H), 5.46-5.50 (m, 1H), 6.15 (d, *J* = 9.6 Hz, 1H), 6.28 (d, *J* = 2.2 Hz, 1H), 6.41 (dd, *J* = 1.6 Hz, 2.2 Hz, 1H), 8.01(dd, *J* = 0.6 Hz, 9.0 Hz, 1H)	ppm 16.74, 17.73, 25.67, 26.24, 39.50, 55.78, 65.72, 92.70, 95.81, 104.29, 110.81, 118.50, 123.59, 131.99, 139.05, 142.15, 155.29, 156.86, 161.64, 163.62	328 (M+)	$C_{20}H_{24}O_4$
5m-1	White powder	147-148	1712 (>C=O), 1613	δ 3.86 (s, OCH_3), 3.89 (s, OCH_3), 6.15 (d, *J* = 9.6 Hz, 1H), 6.28 (d, *J* = 2.2 Hz, 1H), 6.42 (d, *J* = 2.2 Hz, 1H), 7.97 (dd, *J* = 0.6 Hz, 9.6 Hz, 1H)	ppm 55.82, 55.96, 92.81, 94.84, 104.03, 110.97, 138.75, 156.84, 156.99, 161.54, 163.71	206 (M+)	$C_{11}H_{10}O_4$
5m-2	White powder	77-79	1724 (>C=O)	δ 1.60 (s, CH_3), 1.68 (s, CH_3), 1.70 (s, CH_3), 2.06-2.14 (m, -CH_2CH_2-), 4.94 (d, *J* = 6.8Hz, 2H), 5.05-5.09 (m, 1H), 5.52-5.55 (m, 1H), 6.27 (d, *J* = 9.7 Hz, 1H), 6.96 (dd, *J* = 1.2 Hz, 2.4 Hz, 1H), 7.16 (s, 1H), 7.60 (d, *J* = 2.3Hz, 1H), 8.17 (d, *J* = 9.8 Hz, 1H)	ppm 16.68, 17.71, 25.68, 26.22, 39.50, 69.77, 94.24, 105.07, 107.55, 112.58, 114.23, 118.88, 123.50, 132.04, 139.61, 143.05, 144.89, 148.99, 152.68, 158.14, 161.29	338 (M+)	$C_{21}H_{22}O_4$
5m-3	White powder	149-150	1750 (>C=O), 1718, 1622	δ 4.17 (s, OCH_3), 4.18 (s, OCH_3), 6.30 (d, *J* = 9.8 Hz, 1H), 7.00 (d, *J* = 2.3 Hz, 1H), 7.63 (d, *J* = 2.3 Hz, 1H), 8.13 (d, *J* = 9.9 Hz, 1H)	ppm 60.87, 61.75, 105.10, 107.69, 112.92, 114.84, 143.73, 144.31, 150.04, 160.48	246 (M+)	$C_{13}H_{10}O_5$

Table S2. Common synthesis method of coumarin derivatives.

Compound	Material form	Yield (%)	IR (KBr, cm^{-1})	^{1}H-NMR (400 MHz, DMSO$_{d6}$)	^{13}C-NMR (100 MHz, DMSO$_{d6}$)	^{19}F-NMR (470 MHz, $CDCl_3$)	MS *m/z*	CHN analysis
3a	Yellow powder	72	3554, 3111, 1705 (>C=O)	δ 6.74 (s, 1H), 6.92 (d, *J* = 8.0 Hz, 1H), 7.05-7.09 (m, 1H), 9.65 (bs, OH), 10.57 (bs, OH)	ppm 105.95, 111.70(q, $^3J_{CF}$ = 6.0 Hz), 113.10, 115.21, 117.67, 121.78(q, $^1J_{CF}$ = 274.0 Hz), 123.15, 125.89, 132.89, 139.55, 140.16(q, $^2J_{CF}$ = 31.0Hz), 144.03, 150.52, 158.78	δ 63.41 (s, 3F) ppm	366 (M+)	$C_{20}H_{21}F_3O_3$
3b	Yellow powder	66	3554, 3111, 1705 (>C=O), 1614	δ 6.78 (s, 1H), 6.93 (s, 1H), 7.00 (s, 1H), 12.51 (bs, 2OH)	ppm 95.22, 96.29, 99.51, 110.57(q, $^3J_{CF}$ = 8.0 Hz), 121.75(q, $^1J_{CF}$ = 273.0 Hz), 139.50(q, $^2J_{CF}$ = 33.0 Hz), 155.82, 156.98, 159.02, 162.32	δ 61.85 (s, 3F) ppm	366 (M+)	$C_{20}H_{21}F_3O_3$

Table 3. Common synthesis method of geranyloxy coumarin derivative.

Compound	Material form	Yield (%)	m.p. (°C)	IR (KBr, cm^{-1})	^{1}H-NMR (400 MHz, $CDCl_3$)	^{13}C-NMR (100 MHz, $CDCl_3$)	^{19}F-NMR (470 MHz, $CDCl_3$)	MS *m/z*	CHN analysis
5c	White solid	90	62–63	ν 3077 (Aromatic C-H), 3030 (Aromatic C-H), 2974 (Aliphatic C-H), 2914 (Aliphatic C-H), 2894 (Aliphatic C-H), 1730 (Carbonyl (ester -C=O)), 1609 (C=C bond), 1556, 1516, 1452, 1427, 1400, 1351, 1275, 1215, 1192, 1166, 1137, 1014, 999, 958, 872, 818, 781, 715, 649, 626	δ 1.57 (s, 3H, CH_3), 1.62 (s, 3H, CH_3), 1.73 (s, 3H, CH_3), 2.03–2.13 (m, 4H, -CH_2CH_2-), 4.59 (d, *J* = 4.0 Hz, -OCH_2-), 5.01–5.06 (m, 1H, =CH), 5.40–5.45 (m, 1H, =CH), 6.57 (s, 1H, H-3), 6.83 (d, *J* = 2.5 Hz, 1H, H-8), 6.88 (dd, *J* = 2.5 Hz, 9.0 Hz, 1H, H-6), 7.56–7.59 (m, 1H, H-5) ppm	δ 16.79 (CH_3), 17.70 (CH_3), 25.65 (CH_3), 26.20 (CH_2), 39.50 (CH_2), 65.67 (CH_2), 102.13 (CH), 106.88 (CH), 112.04 (q, $^3J_{CF}$ = 5.7 Hz), 113.98 (C), 121.63 (q, $^1J_{CF}$ = 273.8 Hz, CF_3), 123.00 (CH), 123.54 (CH), 126.24 (q, $^4J_{CF}$ = 2.0 Hz), 132.02 (C), 141.61 (q, $^2J_{CF}$ = 32.7 Hz), 142.78 (C), 156.31 (C), 159.49 (C), 162.86 (C) ppm	δ 63.62 (s, 3F) ppm	366 (M^+)	$C_{20}H_{21}F_3O_3$
5d	White solid	93	66-67	3082 (Aromatic C-H), 3053 (Aromatic C-H), 2972 (Aliphatic C-H), 2896 (Aliphatic C-H), 2879 (Aliphatic C-H), 2849 (Aliphatic C-H), 2833 (Aliphatic C-H), 1728	δ 1.60 (s, 3H, CH_3), 1.66 (s, 3H, CH_3), 1.75 (s, 3H, CH_3), 2.06-2.15 (m, 4H, -CH_2CH_2-), 4.59 (d, *J* = 6.7 Hz, 2H, -CH_2-), 5.05-5.09 (m, 1H, =CH), 5.44-5.48 (m, 1H, =CH), 6.24 (d, *J* = 9.5 Hz, 1H, H-3), 6.81 (d, *J* = 2.5 Hz, 1H, H-6), 6.84 (dd, *J* = 2.4 Hz, 8.4 Hz, 1H, H-8), 7.36 (d, *J* = 8.6	δ 16.37 (CH_3), 17.31 (CH_3), 25.26 (CH_3), 25.82 (CH_2), 39.11 (CH_2), 65.08 (CH_2), 101.18 (CH), 112.01 (CH), 112.56 (C), 112.84 (CH), 117.99 (CH), 123.20 (CH), 128.26 (CH), 131.57 (C), 141.98 (C), 143.04 (CH), 155.47		298 (M+)	$C_{19}H_{22}O_3$

				(Carbonyl (ester -C=O)), 1611 (C=C bond), 1507, 1452, 1430, 1403, 1369, 1348, 1280, 1234, 1201, 1165, 1126, 1103, 1022, 990, 889, 852, 830, 776, 760	Hz, 1H, H-5), 7.63 (d, *J* = 9.5 Hz, 1H, H-4)	(C), 160.90 (C), 161.74 (C) ppm			
5e	White solid	91	54-55	3078 (Aromatic C-H), 3028 (Aromatic C-H), 2964 (Aliphatic C-H), 2917 (Aliphatic C-H), 2856 (Aliphatic C-H), 1726 (Carbonyl (ester -C=O)), 1617 (C=C bond), 1508, 1441, 1420,1390, 1345, 1278, 1257, 1199, 1154, 1134, 1070, 992, 982, 843, 825	δ 1.57 (s, 3H, CH_3), 1.63 (s, 3H, CH_3), 1.73 (s, 3H, CH_3), 2.03-2.13 (m, 4H, -CH_2CH_2-), 2.37 (d, *J* = 1.1 Hz, 3H, CH_3), 4.57 (d, *J* = 6.5 Hz, 2H, -CH_2-), 5.03-5.07 (m, 1H, =CH), 5.42-5.46 (m, 1H, =CH), 6.10 (q, *J* = 1,2 Hz, 2.4 Hz, 1H, H-3), 6.79 (d, *J* = 2.5 Hz, 8.8 Hz, 1H, H-8), 6.84 (d, *J* = 2.5 Hz, 1H, H-6), 7.46 (dd, *J* = 2.6 Hz, 8.8 Hz, 1H, H-5)	δ 16.79 (CH_3), 17.73 (CH_3), 18.70 (CH_3), 25.68 (CH_3), 26.24 (CH_2), 39.53 (CH_2), 65.44 (CH_2), 101.59 (CH), 111.85 (CH), 112.94 (C), 113.47 (CH), 118.45 (CH), 123.63 (CH), 125.46 (CH), 131.97 (C), 142.35 (C), 152.61 (C), 155.25 (C), 161.41 (C), 161.95 (C) ppm		312 (M+)	$C_{20}H_{24}O_3$
5f	Light yellow solid	93	136-137	3076 (Aromatic C-H), 3027 (Aromatic C-H), 2960 (Aliphatic C-H), 2909 (Aliphatic C-H), 2851 (Aliphatic C-H), 1720 (Carbonyl (ester -C=O)), 1604 (C=C bond), 1600 (C=C bond), 1548, 1505, 1453, 1378, 1354, 1262, 1257, 1208, 1169, 1142, 1077, 1008, 946, 873, 818, 753, 583;	δ 1.60 (s, 3H, CH_3), 1.66 (s, 3H, CH_3), 1.76 (s, 3H, CH_3), 2.06-2.17 (m, 4H, -CH_2CH_2-), 2.54 (s, 3H, CH_3), 4.60 (d, *J* = 6.6 Hz, -CH_2-), 5.05-5.09 (m, 1H, =CH), 5.43-5.48 (m, 1H, =CH), 6.82 (d, *J* = 2.5 Hz, 1H, H-8), 6.90 (dd, *J* = 2.5 Hz, 8.8 Hz, 1H, H-6), 7.51 (d, *J* = 9.0 Hz, 1H, H-5)	δ 16.12 (CH_3), 16.74 (CH_3), 17.66 (CH_3), 25.62 (CH_3), 26.18 (CH_2), 39.46 (CH_2), 65.50 (CH_2), 101.44 (CH), 113.10 (CH), 113.56 (C), 117.60 (CH), 118.24 (CH), 123.54 (CH), 125.74 (CH), 131.93 (C), 142.46 (C), 147.96 (C), 153.04 (C), 157.47 (C), 161.82 (C) ppm		346 (M+)	$C_{20}H_{23}ClO_3$
5g	White solid	90	95-96	3078 (Aromatic C-H), 3003 Aromatic C-H), 2965 (Aliphatic C-H), 2916 (Aliphatic C-H), 2856 (Aliphatic C-H), 2854 (Aliphatic C-H), 1728 (Carbonyl (ester -C=O)), 1609 (C=C bond), 1494, 1414, 1388, 1378, 1320, 1274, 1205, 1157, 1083, 1047, 982, 883, 829	δ 1.59 (s, 3H, CH_3), 1.65 (s, 3H, CH_3), 1.77 (s, 3H, CH_3), 2.06-2.15 (m, 4H, -CH_2CH_2-), 2.38 (d, *J* = 1.2 Hz, 3H, CH_3), 4.69 (d, *J* = 6.4Hz, 2H, -CH_2-), 5.04-5.08 (m, 1H, =CH), 5.44-5.48 (m, 1H, =CH), 6.16 (dd, *J* = 1.4 Hz, 2.6 Hz, 1H, H-3), 6.83 (s, 1H), 7.56 (s, 1H)	δ 16.89 (CH_3), 17.73 (CH_3), 18.65 (CH_3), 25.65 (CH_3), 26.19 (CH_2), 39.50 (CH_2), 66.63 (CH_2), 101.57 (CH), 112.74 (CH), 113.65 (C), 118.03 (CH), 119.34 (C), 123.54(CH), 125.32 (CH), 132.01 (C), 142.66 (C), 151.70 (C), 153.52 (C), 156.93 (C), 160.79 (C) ppm		346 (M+)	$C_{20}H_{23}ClO_3$

5h	White solid	92	104-105	3054 (Aromatic C-H), 3036 (Aromatic C-H), 2965 (Aliphatic C-H), 2909 (Aliphatic C-H), 2851 (Aliphatic C-H), 1707 (Carbonyl (ester -C=O)), 1606 (C=C bond), 1503, 1450, 1443, 1429, 1365, 1272, 11220, 1178, 1123, 1105, 1012, 990, 941, 827, 784, 691, 630	δ 1.59 (s, 3H, CH_3), 1.66 (s, 3H, CH_3), 1.75 (s, 3H, CH_3), 2.05-2.15 (m, 4H, -CH_2CH_2-), 4.60 (d, *J* = 8.0 Hz, 2H, -CH_2-), 5.05-5.09 (m, 1H, =CH), 5.44-5.48 (m, 1H, =CH), 6.84-6.87 (m, 2H, H-6 and H-8), 7.34-7.44 (m, 4H), 7.65-7.68 (m, 2H), 7.74 (s, 1H, H-4)	δ 16.76 (CH_3), 17.69 (CH_3), 25.64 (CH_3), 26.20 (CH_2), 39.48 (CH_2), 65.47 (CH_2), 101.12 (CH), 113.19 (CH), 113.39 (CH), 118.38 (CH), 123.58 (C), 124.63 (CH), 128.36 (CH), 128.39 (CH), 128.74 (CH), 131.94 (C), 135.03 (C), 140.06 (C), 142.34 (CH), 155.23 (C), 160.95 (C), 161.88 (C) ppm		374 (M+)	$C_{25}H_{26}O_3$
5i	White solid	92	95-96	3067 (Aromatic C-H), 3050 (Aromatic C-H), 2958 (Aliphatic C-H), 2911 (Aliphatic C-H), 2870 (Aliphatic C-H), 2840 (Aliphatic C-H), 1704 (Carbonyl (ester -C=O)), 1566 (C=C bond), 1490, 1443, 1385, 1276, 1175, 1171, 1110, 1017, 922, 882, 816, 706	δ 1.60 (s, 3H, CH_3), 1.66 (s, 3H, CH_3), 1.74 (s, 3H, CH_3), 2.06-2.16 (m, 4H, -CH_2CH_2-), 4.56 (d, *J* = 6.6 Hz, 2H, -CH_2-), 5.05-5.10 (m, 1H, =CH), 5.45-5.50 (m, 1H, =CH), 6.42 (d, *J* = 9.4 Hz, 1H, H-3), 6.92 (d, *J* = 2.9 Hz, 1H, H-5), 7.12 (dd, *J* = 2.9 Hz, 9.0 Hz, 1H, H-7), 7.26 (d, *J* = 9.0 Hz, 1H, H-8), 7.64 (d, *J* = 9.5 Hz, 1H, H-4)	δ 16.74 (CH_3), 17.73 (CH_3), 25.70 (CH_3), 26.25 (CH_2), 39.53 (CH_2), 65.57 (CH_2), 111.08 (CH), 117.02 (CH), 117.83(CH), 118.90 (CH), 119.15 (CH), 120.15 (C), 123.65, 131.95(C), 141.96 (C), 143.28 (CH), 148.41 (C), 155.34 (C), 161.05 (C) ppm		298 (M+)	$C_{19}H_{22}O_3$
5j	White solid	89	57-58	3040 (Aromatic C-H), 2965 (aliphatic C-H), 2925 (aliphatic C-H), 2884 (aliphatic C-H), 1712(C=O), 1673, 1571, 1493, 1428, 1386, 1275, 1238 (C-O), 1167, 990, 926, 838	δ 1.60 (s, 3H, CH_3), 1.67 (s, 3H, CH_3), 1.77 (s, 3H, CH_3), 2.06-2.17 (m, 4H, -CH_2CH_2-), 2.41 (d, *J* = 1.2 Hz, 3H, CH_3), 4.59 (d, *J* = 6.6 Hz, 2H, -CH_2-), 5.06-5.10 (m, 1H, =CH), 5.47-5.51 (m, 1H, =CH), 6.30 (q, *J* = 1.4 Hz, 2.6 Hz, 1H, H-3), 7.04 (d, *J* = 2.9 Hz, 1H, H-5), 7.13 (dd, *J* = 2.9 Hz, 9.0 Hz, 1H, H-7), 7.27 (d, *J* = 9.0 Hz, 1H, H-8)	δ 16.74 (CH_3), 17.72 (CH_3), 18.74 (CH_3), 25.68 (CH_3), 26.27 (CH_2), 39.55 (CH_2), 65.56 (CH_2), 108.82 (CH), 115.44 (CH), 117.89 (CH), 119.00 (CH), 119.32 (C), 120.43 (CH), 123.64 (CH), 131.96 (C), 141.96 (C), 147.84 (C), 152.02 (C), 155.20 (C), 161.03 (C) ppm		312 (M+)	$C_{20}H_{24}O_3$
5k	White solid	86	63-64	3065 (Aromatic C-H), 2937(Aliphatic C-H), 2917 (Aliphatic C-H), 2854 (Aliphatic C-H), 1708 (Carbonyl (ester -C=O)), 1612 (C=C bond), 1562, 1520, 1430, 1384, 1280, 1231, 1164, 984, 822	δ 1.59 (s, 6H, 2CH_3), 1.64 (s, 6H, 2CH_3), 1.77 (s, 6H, 2CH_3), 2.06-2.15 (m, 8H, 2(-CH_2CH_2-)), 2.37 (d, *J* = 1.2 Hz, 3H, CH_3), 4.67 (d, *J* = 6.4 Hz, 4H, 2(-CH_2-)), 5.04-5.08 (m, 2H, 2(=CH)), 5.44-5.48 (m, 2H, 2(=CH)), 6.15 (dd, *J* = 1.4 Hz, 2.64 Hz, 1H, H-3), 6.83 (s, 1H, H-5), 7.55 (s, 1H, H-8)	δ 17.04 (CH_3), 17.17 (CH_3), 18.01 (CH_3), 19.14 (CH_3), 25.95 (CH_3), 26.52 (CH_3), 26.59 (CH_3), 39.81 (CH_2), 39.86 (CH_2), 66.63 (CH_2), 67.16 (CH_2), 101.75 (CH), 108.76 (CH), 112.37 (C), 112.65 (CH), 119.06 (CH), 119.92 (C), 123.96 (CH), 132.22 (C), 141.50 (C), 141.91 (C), 145.76 (C), 149.76 (C), 152.70 (C),		464 (M+)	$C_{30}H_{40}O_4$

						153.07 (C), 161.95 (C) ppm			
5l	White solid	87	72-73	3086 (Aromatic C-H), 3052 (Aromatic C-H), 2975 (Aliphatic C-H), 2917 (Aliphatic C-H), 2885 (Aliphatic C-H), 1745 (Carbonyl (ester -C=O)), 1638 (C=C bond), 1585, 1503, 1468, 1446, 1413, 1390, 1331, 1319, 1225, 1218, 1164, 1122, 996, 950, 938, 900, 864, 790, 761, 603	δ 1.57 (s, 3H, CH_3), 1.62 (s, 3H, CH_3), 1.73 (s, 3H, CH_3), 2.03-2.13 (m, 4H, -CH_2CH_2-), 4.61 (d, *J* = 8.0 Hz, 2H, -CH_2-), 5.02-5.06 (m, 1H, =CH), 5.46-5.51 (m, 1H, =CH), 6.79 (s, 1H, H-4), 7.20-7.28 (m, 2H, H6 and H-8), 7.32-7.37 (m, 2H, H-5 and H-7)	δ 16.82 (CH_3), 17.72 (CH_3), 25.66 (CH_3), 26.17 (CH), 39.50 (CH), 66.24 (CH), 113.69 (CH), 116.29 (CH), 117.96 (C), 119.82(CH), 123.59 (C), 124.64(CH), 126.37 (CH), 128.33 (CH), 132.00 (CH), 142.51 (C), 143.69 (C), 149.53 (C), 157.77 (C) ppm		298 (M+)	$C_{19}H_{22}O_3$
5oa	Colorless liquid	11		2966 (Aliphatic C-H), 2917 (Aliphatic C-H), 2854 (Aliphatic C-H), 1772 (C=O), 1689 (C=O), 1611, 1461, 1288 (C-O)	δ 1.47 (s, 6H, 2CH_3), 1.55 (s, 6H, 2CH_3), 1.59 (s, 6H, 2CH_3), 1.72-1.84 (m, 8H, 2(-CH_2CH_2-)), 2.70-2.80 (m, 2H, -CH_2-), 2.82-2.89 (m, 2H, -CH_2-), 4.86-4.95 (m, 4H, 4(=CH)), 7.14-7.17 (m, 1H, H-6), 7.21-7.25 (m, 1H, H-5), 7.58-7.63 (m, 1H, H-8), 7.89-7.93 (m, 1H, H-7)	δ 16.21 (CH_3), 17.52 (CH_3), 25.49 (CH_3), 26.33 (CH), 37.62 (CH), 39.65 (CH), 62.24 (C), 116.89 (CH), 117.49 (C), 119.53 (CH), 123.78 (CH), 124.66 (CH), 126.55 (CH), 131.32 (C), 136.82 (C), 140.59 (C), 154.89 (C), 170.56 (C), 194.66 (C) ppm		434 (M+)	
5ob	Colorless liquid	35		ν 1634(C=O), 1443(C-O)	δ 1.54 (s, 6H, 2CH_3), 1.58 (s, 6H, 2CH_3), 1.62 (s, 6H, 2CH_3), 1.84–1.98 (m, 8H, 2(-CH_2CH_2-)), 2.25–2.32 (m, 2H, -CH_2-), 2.41–2.48 (m, 2H, -CH_2-), 3.47–3.54 (m, 1H, -C-H), 4.98–5.03 (m, 2H, 2(=C-H)), 5.06–5.11(m, 2H, 2(=C-H)), 6.86–6.90 (m, 1H), 6.95–6.98 (m, 1H), 7.42–7.46 (m, 1H), 7.76–7.79 (dd, *J* = 1.8 Hz, 8.2 Hz, 1H), 12.66 (s, 1H, OH, D_2O exch.) ppm	δ 16.10 (CH_3), 17.67 (CH_3), 25.65 (CH_3), 26.54 (CH_2), 30.60 (CH_2), 39.74 (CH_2), 46.39 (CH), 118.56 (CH), 118.68 (CH), 119.65 (C), 121.10 (CH), 124.11 (CH), 130.24 (CH), 131.45 (C), 136.20 (CH), 137.59 (C), 162.93 (C), 210.28 (-C=O) ppm		408 (M^+)	
5oc	White powder	18	47-48	2923 (Aliphatic C-H), 1718 (C=O), 1620, 1371, 1235 (C-O), 1182 (C-O), 1104 (C-O), 923, 817, 764, 751, 500	δ 1.62 (s, 3H, CH_3), 1.69 (s, 3H, CH_3), 1.77 (s, 3H, CH_3), 2.08-2.18 (m, 4H, -CH_2CH_2-), 4.71 (d, *J* = 6.7 Hz, 2H, -CH_2-), 5.08-5.12 (m, 1H, =CH), 5.49-5.53 (m, 1H, =CH), 5.69 (s, 1H, H-3), 7.25-7.33 (m, H6 and H-8), 7.53-7.57 (m, 1H, H-7), 7.84 (dd, J=2.2Hz, 5.8Hz, 1H, H-5)	δ 16.82 (CH_3), 17.75 (CH_3), 25.70 (CH_3), 26.19 (CH), 39.50 (CH), 66.27 (CH), 90.64 (CH), 115.92 (C), 116.75 (CH), 117.06 (CH), 123.19 (CH), 123.46 (CH), 123.83 (CH), 132.13 (CH), 132.31 (C), 143.74 (C), 153.35 (C), 163.15 (C), 165.61 (C) ppm		298 (M+)	$C_{19}H_{22}O_3$

Supplementary Materials

1. Compounds separated by lime

(E)-5-((3,7-Dimethylocta-2,6-dien-1-yl)oxy)-7-methoxy-2H-chromen-2-one (***5m***): White powder; mp 86-87 °C; IR (KBr, cm^{-1}): 1726 (>C=O), 1606, 1437, 1040; ^{1}H-NMR (400 MHz, $CDCl_3$): δ 1.61 (s, CH_3), 1.68 (s, CH_3), 1.75 (s, CH_3), 2.07-2.16 (m, 4H, -CH_2CH_2-), 3.85 (s, OCH_3), 4.60 (d, *J* = 7.2 Hz, -CH_2-), 5.05-5.11 (m, 1H), 5.46-5.50 (m, 1H), 6.15 (d, *J* = 9.6 Hz, 1H), 6.28 (d, *J* = 2.2 Hz, 1H), 6.41 (dd, *J* = 1.6 Hz, 2.2 Hz, 1H), 8.01(dd, *J* = 0.6 Hz, 9.0 Hz, 1H); ^{13}C-NMR (100 MHz, $CDCl_3$): ppm 16.74, 17.73, 25.67, 26.24, 39.50, 55.78, 65.72, 92.70, 95.81, 104.29, 110.81, 118.50, 123.59, 131.99, 139.05, 142.15, 155.29, 156.86, 161.64, 163.62; MS *m/z* = 328 (M+); CHN analysis ($C_{20}H_{24}O_4$) Theoretical value ; C, 73.15; H, 7.37. Experimental value ; C, 73.14; H, 7.37.

5,7-Dimethoxy-2H-chromen-2-one (***5m-1***): White powder; mp 147-148 ℃; IR (KBr, cm^{-1}): 1712 (>C=O), 1613; ^{1}H-NMR (400 MHz, $CDCl_3$): δ 3.86 (s, OCH_3), 3.89 (s, OCH_3), 6.15 (d, *J* = 9.6 Hz, 1H), 6.28 (d, *J* = 2.2 Hz, 1H), 6.42 (d, *J* = 2.2 Hz, 1H), 7.97 (dd, *J* = 0.6 Hz, 9.6 Hz, 1H) ; ^{13}C-NMR (100 MHz, $CDCl_3$): ppm 55.82, 55.96, 92.81, 94.84, 104.03, 110.97, 138.75, 156.84, 156.99, 161.54, 163.71; MS *m/z* = 206 (M+); CHN analysis ($C_{11}H_{10}O_4$) Theoretical value ; C, 64.08; H, 4.89. Experimental value ; C, 64.05; H, 4.87.

(E)-4-((3,7-dimethylocta-2,6-dien-1-yl)oxy)-7H-furo[3,2-g]chromen-7-one (***5m-2***): White powder; mp 77-79 ℃; IR (KBr, cm^{-1}): 1724 (>C=O); ^{1}H-NMR (400 MHz, $CDCl_3$): δ 1.60 (s, CH_3), 1.68 (s, CH_3), 1.70 (s, CH_3), 2.06-2.14 (m, -CH_2CH_2-), 4.94 (d, *J* = 6.8Hz, 2H), 5.05-5.09 (m, 1H), 5.52-5.55 (m, 1H), 6.27 (d, *J* = 9.7 Hz, 1H), 6.96 (dd, *J* = 1.2 Hz, 2.4 Hz, 1H), 7.16 (s, 1H), 7.60 (d, *J* = 2.3Hz, 1H), 8.17 (d, *J* = 9.8 Hz, 1H); ^{13}C-NMR (100 MHz, $CDCl_3$): ppm 16.68, 17.71, 25.68, 26.22, 39.50, 69.77, 94.24, 105.07, 107.55, 112.58, 114.23, 118.88, 123.50, 132.04, 139.61, 143.05, 144.89, 148.99, 152.68, 158.14, 161.29; MS *m/z* = 338 (M+); CHN analysis ($C_{21}H_{22}O_4$): Theoretical value ; C, 74.54; H, 6.55. Experimental value ; C, 74.53; H, 6.52.

4,9-Dimethoxy-7H-furo[3,2-g]chromen-7-one(or isopimpinellin) (***5m-3***): White powder; mp 149-150 ℃; IR (KBr, cm^{-1}): 1750 (>C=O), 1718, 1622; ^{1}H-NMR (400 MHz, $CDCl_3$): δ 4.17 (s, OCH_3), 4.18 (s, OCH_3), 6.30 (d, *J* = 9.8 Hz, 1H), 7.00 (d, *J* = 2.3 Hz, 1H), 7.63 (d, *J* = 2.3 Hz, 1H), 8.13 (d, *J* = 9.9 Hz, 1H); ^{13}C-NMR (100 MHz, $CDCl_3$): ppm 60.87, 61.75, 105.10, 107.69, 112.92, 114.84, 143.73, 144.31, 150.04, 160.48; MS *m/z* = 246 (M+); CHN analysis ($C_{13}H_{10}O_5$): Theoretical value ; C, 63.42; H, 32.49. Experimental value ; C, 63.41; H, 32.48.

2. Common synthesis method of coumarin derivatives

7,8-Dihydroxy-4-trifluoromethylcoumarin (***3a***): Yellow powder; Yield : 72%; IR (KBr, cm^{-1}): 3554, 3111, 1705 (>C=O), 1614; ^{1}H-NMR (400 MHz, $DMSO_{d6}$): δ 6.74 (s, 1H), 6.92 (d, *J* = 8.0 Hz, 1H), 7.05-7.09 (m, 1H), 9.65 (bs, OH), 10.57 (bs, OH); ^{13}C-NMR (100 MHz, $DMSO_{d6}$): ppm 105.95, 111.70(q, $^{3}J_{CF}$ = 6.0 Hz), 113.10, 115.21, 117.67, 121.78(q, $^{1}J_{CF}$ = 274.0 Hz), 123.15, 125.89,

132.89, 139.55, 140.16(q, $^2J_{CF}$ = 31.0Hz), 144.03, 150.52, 158.78; ^{19}F-NMR (470 MHz, $CDCl_3$): δ 63.41 (s, 3F) ppm; MS *m/z* = 366 (M+); CHN analysis ($C_{20}H_{21}F_3O_3$) Theoretical value ; C, 65.57; H, 5.78. Experimental value ; C, 65.54; H, 5.76.

5,7-Dihydroxy-4-trifluoromethylcoumarin (***3b***): Yellow powder; Yield : 66%; IR (KBr, cm^{-1}): 3554, 3111, 1705 (>C=O), 1614; ^{1}H-NMR (400 MHz, $DMSO_{d6}$): δ 6.78 (s, 1H), 6.93 (s, 1H), 7.00 (s, 1H), 12.51 (bs, 2OH); ^{13}C-NMR (100 MHz, $DMSO_{d6}$): ppm 95.22, 96.29, 99.51, 110.57(q, $^3J_{CF}$ = 8.0 Hz), 121.75(q, $^1J_{CF}$ = 273.0 Hz), 139.50(q, $^2J_{CF}$ = 33.0 Hz), 155.82, 156.98, 159.02, 162.32; ^{19}F-NMR (470 MHz, $CDCl_3$): δ 61.85 (s, 3F) ppm; MS *m/z* = 366 (M+); CHN analysis ($C_{20}H_{21}F_3O_3$) Theoretical value ; C, 65.57; H, 5.78. Experimental value ; C, 65.54; H, 5.76.

3. Common synthesis method of geranyloxy coumarin derivative

(E)-7-((3,7-dimethylocta-2,6-dien-1-yl)oxy)-4-(trifluoromethyl)-2H-chromen-2-one (***5c***): White solid, Yield: 90%; m.p. 62–63 °C; IR (KBr, cm^{-1}): ν 3077 (Aromatic C-H), 3030 (Aromatic C-H), 2974 (Aliphatic C-H), 2914 (Aliphatic C-H), 2894 (Aliphatic C-H), 1730 (Carbonyl (ester -C=O)), 1609 (C=C bond), 1556, 1516, 1452, 1427, 1400, 1351, 1275, 1215, 1192, 1166, 1137, 1014, 999, 958, 872, 818, 781, 715, 649, 626 ; ^{1}H-NMR (400 MHz, $CDCl_3$): δ 1.57 (s, 3H, CH_3), 1.62 (s, 3H, CH_3), 1.73 (s, 3H, CH_3), 2.03–2.13 (m, 4H, -CH_2CH_2-), 4.59 (d, *J* = 4.0 Hz, -OCH_2-), 5.01–5.06 (m, 1H, =CH), 5.40–5.45 (m, 1H, =CH), 6.57 (s, 1H, H-3), 6.83 (d, *J* = 2.5 Hz, 1H, H-8), 6.88 (dd, *J* = 2.5 Hz, 9.0 Hz, 1H, H-6), 7.56–7.59 (m, 1H, H-5) ppm; ^{13}C-NMR (100 MHz, $CDCl_3$): δ 16.79 (CH_3), 17.70 (CH_3), 25.65 (CH_3), 26.20 (CH_2), 39.50 (CH_2), 65.67 (CH_2), 102.13 (CH), 106.88 (CH), 112.04 (q, $^3J_{CF}$ = 5.7 Hz), 113.98 (C), 121.63 (q, $^1J_{CF}$ = 273.8 Hz, CF_3), 123.00 (CH), 123.54 (CH), 126.24 (q, $^4J_{CF}$ = 2.0 Hz), 132.02 (C), 141.61 (q, $^2J_{CF}$ = 32.7 Hz), 142.78 (C), 156.31 (C), 159.49 (C), 162.86 (C) ppm; ^{19}F-NMR (470 MHz, $CDCl_3$): δ 63.62 (s, 3F) ppm; MS *m/z* = 366 (M^+); Anal. Calcd for $C_{20}H_{21}F_3O_3$: C, 65.57; H, 5.78, Found: C, 65.54; H, 5.76.

(E)-7-((3,7-dimethylocta-2,6-dien-1-yl)oxy)-2H-chromen-2-one (***5d***) [46]: White solid; Yield: 93%; mp 66-67 °C; IR (KBr, cm^{-1}): 3082 (Aromatic C-H), 3053 (Aromatic C-H), 2972 (Aliphatic C-H), 2896 (Aliphatic C-H), 2879 (Aliphatic C-H), 2849 (Aliphatic C-H), 2833 (Aliphatic C-H), 1728 (Carbonyl (ester -C=O)), 1611 (C=C bond), 1507, 1452, 1430, 1403, 1369, 1348, 1280, 1234, 1201, 1165, 1126, 1103, 1022, 990, 889, 852, 830, 776, 760; ^{1}H-NMR (400MHz, $CDCl_3$): δ 1.60 (s, 3H, CH_3), 1.66 (s, 3H, CH_3), 1.75 (s, 3H, CH_3), 2.06-2.15 (m, 4H, -CH_2CH_2-), 4.59 (d, *J* = 6.7 Hz, 2H, -CH_2-), 5.05-5.09 (m, 1H, =CH), 5.44-5.48 (m, 1H, =CH), 6.24 (d, *J* = 9.5 Hz, 1H, H-3), 6.81 (d, *J* = 2.5 Hz, 1H, H-6), 6.84 (dd, *J* = 2.4 Hz, 8.4 Hz, 1H, H-8), 7.36 (d, *J* = 8.6 Hz, 1H, H-5), 7.63 (d, *J* = 9.5 Hz, 1H, H-4); ^{13}C-NMR (100MHz, $CDCl_3$): δ 16.37 (CH_3), 17.31 (CH_3), 25.26 (CH_3), 25.82 (CH_2), 39.11 (CH_2), 65.08 (CH_2), 101.18 (CH), 112.01 (CH), 112.56 (C), 112.84 (CH), 117.99 (CH), 123.20 (CH), 128.26 (CH), 131.57 (C), 141.98 (C), 143.04

(CH), 155.47 (C), 160.90 (C), 161.74 (C) ppm; MS *m/z* = 298 (M+); Anal. Calcd for $C_{19}H_{22}O_3$: C, 76.48; H, 7.43, Found: C, 76.45; H, 7.41.

(E)-7-((3,7-dimethylocta-2,6-dien-1-yl)oxy)-4-methyl-2H-chromen-2-one (***5e***) [47]: White solid; Yield: 91%; mp 54-55 °C; IR(KBr, cm^{-1}): 3078 (Aromatic C-H), 3028 (Aromatic C-H), 2964 (Aliphatic C-H), 2917 (Aliphatic C-H), 2856 (Aliphatic C-H), 1726 (Carbonyl (ester -C=O)), 1617 (C=C bond), 1508, 1441, 1420,1390, 1345, 1278, 1257, 1199, 1154, 1134, 1070, 992, 982, 843, 825; ^{1}H-NMR (400MHz, $CDCl_3$): δ 1.57 (s, 3H, CH_3), 1.63 (s, 3H, CH_3), 1.73 (s, 3H, CH_3), 2.03-2.13 (m, 4H, -CH_2CH_2-), 2.37 (d, *J* = 1.1 Hz, 3H, CH_3), 4.57 (d, *J* = 6.5 Hz, 2H, -CH_2-), 5.03-5.07 (m, 1H, =CH), 5.42-5.46 (m, 1H, =CH), 6.10 (q, *J* = 1,2 Hz, 2.4 Hz, 1H, H-3), 6.79 (d, *J* = 2.5 Hz, 8.8 Hz, 1H, H-8), 6.84 (d, *J* = 2.5 Hz, 1H, H-6), 7.46 (dd, *J* = 2.6 Hz, 8.8 Hz, 1H, H-5); ^{13}C-NMR (100MHz, $CDCl_3$): δ 16.79 (CH_3), 17.73 (CH_3), 18.70 (CH_3), 25.68 (CH_3), 26.24 (CH_2), 39.53 (CH_2), 65.44 (CH_2), 101.59 (CH), 111.85 (CH), 112.94 (C), 113.47 (CH), 118.45 (CH), 123.63 (CH), 125.46 (CH), 131.97 (C), 142.35 (C), 152.61 (C), 155.25 (C), 161.41 (C), 161.95 (C) ppm ; MS *m/z* = 312 (M+); Anal. Calcd for $C_{20}H_{24}O_3$: C, 76.89; H, 7.74, Found: C, 76.88; H, 7.72.

(E)-3-chloro-7-((3,7-dimethylocta-2,6-dien-1-yl)oxy)-4-methyl-2H-chromen-2-one (***5f***) [48]: Light yellow solid; Yield: 93%; mp 136-137 °C; IR (KBr, cm^{-1}): 3076 (Aromatic C-H), 3027 (Aromatic C-H), 2960 (Aliphatic C-H), 2909 (Aliphatic C-H), 2851 (Aliphatic C-H), 1720 (Carbonyl (ester -C=O)), 1604 (C=C bond), 1600 (C=C bond), 1548, 1505, 1453, 1378, 1354, 1262, 1257, 1208, 1169, 1142, 1077, 1008, 946, 873, 818, 753, 583; ^{1}H-NMR (400MHz, $CDCl_3$): δ 1.60 (s, 3H, CH_3), 1.66 (s, 3H, CH_3), 1.76 (s, 3H, CH_3), 2.06-2.17 (m, 4H, -CH_2CH_2-), 2.54 (s, 3H, CH_3), 4.60 (d, *J* = 6.6 Hz, -CH_2-), 5.05-5.09 (m, 1H, =CH), 5.43-5.48 (m, 1H, =CH), 6.82 (d, *J* = 2.5 Hz, 1H, H-8), 6.90 (dd, *J* = 2.5 Hz, 8.8 Hz, 1H, H-6), 7.51 (d, *J* = 9.0 Hz, 1H, H-5); ^{13}C-NMR (100MHz, $CDCl_3$): δ 16.12 (CH_3), 16.74 (CH_3), 17.66 (CH_3), 25.62 (CH_3), 26.18 (CH_2), 39.46 (CH_2), 65.50 (CH_2), 101.44 (CH), 113.10 (CH), 113.56 (C), 117.60 (CH), 118.24 (CH), 123.54 (CH), 125.74 (CH), 131.93 (C), 142.46 (C), 147.96 (C), 153.04 (C), 157.47 (C), 161.82 (C) ppm ; MS *m/z* = 346 (M+); Anal. Calcd for $C_{20}H_{23}ClO_3$: C, 69.26; H, 6.68, Found: C, 69.25; H, 6.67.

(E)-6-chloro-7-((3,7-dimethylocta-2,6-dien-1-yl)oxy)-4-methyl-2H-chromen-2-one (***5g***): White solid; Yield: 90%; mp 95-96 °C; IR (KBr, cm^{-1}): 3078 (Aromatic C-H), 3003 Aromatic C-H), 2965 (Aliphatic C-H), 2916 (Aliphatic C-H), 2856 (Aliphatic C-H), 2854 (Aliphatic C-H), 1728 (Carbonyl (ester -C=O)), 1609 (C=C bond), 1494, 1414, 1388, 1378, 1320, 1274, 1205, 1157, 1083, 1047, 982, 883, 829; ^{1}H-NMR (400MHz, $CDCl_3$): δ 1.59 (s, 3H, CH_3), 1.65 (s, 3H, CH_3), 1.77 (s, 3H, CH_3), 2.06-2.15 (m, 4H, -CH_2CH_2-), 2.38 (d, *J* = 1.2 Hz, 3H, CH_3), 4.69 (d, *J* = 6.4Hz, 2H, -CH_2-), 5.04-5.08 (m, 1H, =CH), 5.44-5.48 (m, 1H, =CH), 6.16 (dd, *J* = 1.4 Hz, 2.6 Hz, 1H, H-3), 6.83 (s, 1H), 7.56 (s, 1H); ^{13}C-NMR (100MHz, $CDCl_3$): δ 16.89 (CH_3), 17.73 (CH_3), 18.65 (CH_3), 25.65 (CH_3), 26.19 (CH_2), 39.50 (CH_2), 66.63 (CH_2), 101.57 (CH), 112.74 (CH), 113.65 (C), 118.03 (CH), 119.34 (C), 123.54(CH), 125.32 (CH), 132.01 (C), 142.66 (C), 151.70 (C), 153.52 (C), 156.93 (C), 160.79 (C) ppm; MS *m/z* = 346 (M+); Anal. Calcd for $C_{20}H_{23}ClO_3$: C, 69.26; H, 6.68, Found: C, 69.24; H, 6.65.

(E)-7-((3,7-dimethylocta-2,6-dien-1-yl)oxy)-3-phenyl-2H-chromen-2-one (***5h***): White solid; Yield: 92%; mp 104-105 °C; IR (KBr, cm^{-1}): 3054 (Aromatic C-H), 3036 (Aromatic C-H), 2965 (Aliphatic C-H), 2909 (Aliphatic C-H), 2851 (Aliphatic C-H), 1707 (Carbonyl (ester -C=O)), 1606 (C=C bond), 1503, 1450, 1443, 1429, 1365, 1272, 11220, 1178, 1123, 1105, 1012, 990, 941, 827, 784, 691, 630; ^{1}H-NMR (400MHz, $CDCl_3$): δ 1.59 (s, 3H, CH_3), 1.66 (s, 3H, CH_3), 1.75 (s, 3H, CH_3), 2.05-2.15 (m, 4H, -CH_2CH_2-), 4.60 (d, *J* = 8.0 Hz, 2H, -CH_2-), 5.05-5.09 (m, 1H, =CH), 5.44-5.48 (m, 1H, =CH), 6.84-6.87 (m, 2H, H-6 and H-8), 7.34-7.44 (m, 4H), 7.65-7.68 (m, 2H), 7.74 (s, 1H, H-4); ^{13}C-NMR (100MHz, $CDCl_3$): δ 16.76 (CH_3), 17.69 (CH_3), 25.64 (CH_3), 26.20 (CH_2), 39.48 (CH_2), 65.47 (CH_2), 101.12 (CH), 113.19 (CH), 113.39 (CH), 118.38 (CH), 123.58 (C), 124.63 (CH), 128.36 (CH), 128.39 (CH), 128.74 (CH), 131.94 (C), 135.03 (C), 140.06 (C), 142.34 (CH), 155.23 (C), 160.95 (C), 161.88 (C) ppm; MS *m/z* = 374 (M+); Anal. Calcd for $C_{25}H_{26}O_3$: C, 80.18; H, 7.00, Found: C, 80.16; H, 6.99.

(E)-6-((3,7-dimethylocta-2,6-dien-1-yl)oxy)-2H-chromen-2-one (***5i***): White solid; Yield: 92%; mp 95-96 °C; IR (KBr, cm^{-1}): 3067 (Aromatic C-H), 3050 (Aromatic C-H), 2958 (Aliphatic C-H), 2911 (Aliphatic C-H), 2870 (Aliphatic C-H), 2840 (Aliphatic C-H), 1704 (Carbonyl (ester -C=O)), 1566 (C=C bond), 1490, 1443, 1385, 1276, 1175, 1171, 1110, 1017, 922, 882, 816, 706; ^{1}H-NMR (400MHz, $CDCl_3$): δ 1.60 (s, 3H, CH_3), 1.66 (s, 3H, CH_3), 1.74 (s, 3H, CH_3), 2.06-2.16 (m, 4H, -CH_2CH_2-), 4.56 (d, *J* = 6.6 Hz, 2H, -CH_2-), 5.05-5.10 (m, 1H, =CH), 5.45-5.50 (m, 1H, =CH), 6.42 (d, *J* = 9.4 Hz, 1H, H-3), 6.92 (d, *J* = 2.9 Hz, 1H, H-5), 7.12 (dd, *J* = 2.9 Hz, 9.0 Hz, 1H, H-7), 7.26 (d, *J* = 9.0 Hz, 1H, H-8), 7.64 (d, *J* = 9.5 Hz, 1H, H-4); ^{13}C-NMR (100MHz, $CDCl_3$): δ 16.74 (CH_3), 17.73 (CH_3), 25.70 (CH_3), 26.25 (CH_2), 39.53 (CH_2), 65.57 (CH_2), 111.08 (CH), 117.02 (CH), 117.83(CH), 118.90 (CH), 119.15 (CH), 120.15 (C), 123.65, 131.95(C), 141.96 (C), 143.28 (CH), 148.41 (C), 155.34 (C), 161.05 (C) ppm; MS *m/z* = 298 (M+); Anal. Calcd for $C_{19}H_{22}O_3$: C, 76.48; H, 7.43, Found: C, 76.46; H, 7.42.

(E)-6-((3,7-dimethylocta-2,6-dien-1-yl)oxy)-4-methyl-2H-chromen-2-one (***5j***) [46, 47]: White solid; Yield: 89%; mp 57-58 °C; IR (KBr, cm^{-1}): 3040 (Aromatic C-H), 2965 (aliphatic C-H), 2925 (aliphatic C-H), 2884 (aliphatic C-H), 1712(C=O), 1673, 1571, 1493, 1428, 1386, 1275, 1238 (C-O), 1167, 990, 926, 838; ^{1}H-NMR (400MHz, $CDCl_3$): δ 1.60 (s, 3H, CH_3), 1.67 (s, 3H, CH_3), 1.77 (s, 3H, CH_3), 2.06-2.17 (m, 4H, -CH_2CH_2-), 2.41 (d, *J* = 1.2 Hz, 3H, CH_3), 4.59 (d, *J* = 6.6 Hz, 2H, -CH_2-), 5.06-5.10 (m, 1H, =CH), 5.47-5.51 (m, 1H, =CH), 6.30 (q, *J* = 1.4 Hz, 2.6 Hz, 1H, H-3), 7.04 (d, *J* = 2.9 Hz, 1H, H-5), 7.13 (dd, *J* = 2.9 Hz, 9.0 Hz, 1H, H-7), 7.27 (d, *J* = 9.0 Hz, 1H, H-8); ^{13}C-NMR (100MHz, $CDCl_3$): δ 16.74 (CH_3), 17.72 (CH_3), 18.74 (CH_3), 25.68 (CH_3), 26.27 (CH_2), 39.55 (CH_2), 65.56 (CH_2), 108.82 (CH), 115.44 (CH), 117.89 (CH), 119.00 (CH), 119.32 (C), 120.43 (CH), 123.64 (CH), 131.96 (C), 141.96 (C), 147.84 (C), 152.02 (C), 155.20 (C), 161.03 (C) ppm; MS *m/z* = 312 (M+); Anal. Calcd for $C_{20}H_{24}O_3$: C, 76.89; H, 7.74, Found: C, 76.86; H, 7.75.

6-(((E)-3,7-dimethylocta-2,6-dien-1-yl)oxy)-7-(((Z)-3,7-dimethylocta-2,6-dien-1-yl)oxy)-4-methyl-2H-chrom en-2-one (***5k***): White solid; Yield: 86%; mp 63-64 °C; IR (KBr, cm^{-1}): 3065 (Aromatic C-H), 2937(Aliphatic C-H), 2917 (Aliphatic C-H), 2854 (Aliphatic C-H), 1708 (Carbonyl (ester -C=O)), 1612 (C=C bond), 1562, 1520, 1430, 1384, 1280, 1231, 1164, 984, 822; ^{1}H-NMR (400MHz, $CDCl_3$): δ 1.59 (s, 6H, 2CH_3), 1.64 (s, 6H, 2CH_3), 1.77 (s, 6H, 2CH_3), 2.06-2.15 (m, 8H, 2(-CH_2CH_2-)), 2.37 (d, *J* = 1.2

Hz, 3H, CH_3), 4.67 (d, *J* = 6.4 Hz, 4H, 2(-CH_2-)), 5.04-5.08 (m, 2H, 2(=CH)), 5.44-5.48 (m, 2H, 2(=CH)), 6.15 (dd, *J* = 1.4 Hz, 2.64 Hz, 1H, H-3), 6.83 (s, 1H, H-5), 7.55 (s, 1H, H-8); ^{13}C-NMR (100MHz, $CDCl_3$): δ 17.04 (CH_3), 17.17 (CH_3), 18.01 (CH_3), 19.14 (CH_3), 25.95 (CH_3), 26.52 (CH_3), 26.59 (CH_3), 39.81 (CH_2), 39.86 (CH_2), 66.63 (CH_2), 67.16 (CH_2), 101.75 (CH), 108.76 (CH), 112.37 (C), 112.65 (CH), 119.06 (CH), 119.92 (C), 123.96 (CH), 132.22 (C), 141.50 (C), 141.91 (C), 145.76 (C), 149.76 (C), 152.70 (C), 153.07 (C), 161.95 (C) ppm; MS *m/z* = 464 (M+); Anal. Calcd for $C_{30}H_{40}O_4$: C, 77.55; H, 8.68, Found: C, 77.54; H, 8.66.

(E)-3-((3,7-dimethylocta-2,6-dien-1-yl)oxy)-2H-chromen-2-one (***5l***) [46]: White solid; Yield: 87%; mp 72-73 °C; IR (KBr, cm^{-1}): 3086 (Aromatic C-H), 3052 (Aromatic C-H), 2975 (Aliphatic C-H), 2917 (Aliphatic C-H), 2885 (Aliphatic C-H), 1745 (Carbonyl (ester -C=O)), 1638 (C=C bond), 1585, 1503, 1468, 1446, 1413, 1390, 1331, 1319, 1225, 1218, 1164, 1122, 996, 950, 938, 900, 864, 790, 761, 603; ^{1}H-NMR (400MHz, $CDCl_3$): δ 1.57 (s, 3H, CH_3), 1.62 (s, 3H, CH_3), 1.73 (s, 3H, CH_3), 2.03-2.13 (m, 4H, -CH_2CH_2-), 4.61 (d, *J* = 8.0 Hz, 2H, -CH_2-), 5.02-5.06 (m, 1H, =CH), 5.46-5.51 (m, 1H, =CH), 6.79 (s, 1H, H-4), 7.20-7.28 (m, 2H, H6 and H-8), 7.32-7.37 (m, 2H, H-5 and H-7); ^{13}C-NMR (100MHz, CDCl3): δ 16.82 (CH_3), 17.72 (CH_3), 25.66 (CH_3), 26.17 (CH), 39.50 (CH), 66.24 (CH), 113.69 (CH), 116.29 (CH), 117.96 (C), 119.82(CH), 123.59 (C), 124.64(CH), 126.37 (CH), 128.33 (CH), 132.00 (CH), 142.51 (C), 143.69 (C), 149.53 (C), 157.77 (C) ppm; MS *m/z* = 298 (M+); Anal. Calcd for $C_{19}H_{22}O_3$: C, 76.48; H, 7.43, Found: C, 76.44; H, 7.42.

3,3-Bis((E)-3,7-dimethylocta-2,6-dien-1-yl)chromane-2,4-dione(***5oa***) [49]:
Colorless liquid; Yield: 11%; IR (KBr, cm^{-1}): 2966 (Aliphatic C-H), 2917 (Aliphatic C-H), 2854 (Aliphatic C-H), 1772 (C=O), 1689 (C=O), 1611, 1461, 1288 (C-O); ^{1}H-NMR (400MHz, $CDCl_3$): δ 1.47 (s, 6H, 2CH_3), 1.55 (s, 6H, 2CH_3), 1.59 (s, 6H, 2CH_3), 1.72-1.84 (m, 8H, 2(-CH_2CH_2-)), 2.70-2.80 (m, 2H, -CH_2-), 2.82-2.89 (m, 2H, -CH_2-), 4.86-4.95 (m, 4H, 4(=CH)), 7.14-7.17 (m, 1H, H-6), 7.21-7.25 (m, 1H, H-5), 7.58-7.63 (m, 1H, H-8), 7.89-7.93 (m, 1H, H-7); ^{13}C-NMR (100MHz, $CDCl_3$): δ 16.21 (CH_3), 17.52 (CH_3), 25.49 (CH_3), 26.33 (CH), 37.62 (CH), 39.65 (CH), 62.24 (C), 116.89 (CH), 117.49 (C), 119.53 (CH), 123.78 (CH), 124.66 (CH), 126.55 (CH), 131.32 (C), 136.82 (C), 140.59 (C), 154.89 (C), 170.56 (C), 194.66 (C) ppm; MS *m/z* = 434 (M+).

(E)-2-((E)-3,7-dimethylocta-2,6-dien-1-yl)-1-(2-hydroxyphenyl)-5,9- dimethyldeca -4,8-dien-1-one(***5ob***): Colorless liquid, Yield : 35%; IR (KBr, cm^{-1}): ν 1634(C=O), 1443(C-O); ^{1}H-NMR (400MHz, $CDCl_3$): δ 1.54 (s, 6H, 2CH_3), 1.58 (s, 6H, 2CH_3), 1.62 (s, 6H, 2CH_3), 1.84–1.98 (m, 8H, 2(-CH_2CH_2-)), 2.25–2.32 (m, 2H, -CH_2-), 2.41–2.48 (m, 2H, -CH_2-), 3.47–3.54 (m, 1H, -C-H), 4.98–5.03 (m, 2H, 2(=C-H)), 5.06–5.11(m, 2H, 2(=C-H)), 6.86–6.90 (m, 1H), 6.95–6.98 (m, 1H), 7.42–7.46 (m, 1H), 7.76–7.79 (dd, *J* = 1.8 Hz, 8.2 Hz, 1H), 12.66 (s, 1H, OH, D_2O exch.) ppm; ^{13}C-NMR (100MHz, $CDCl_3$): δ 16.10 (CH_3), 17.67 (CH_3), 25.65 (CH_3), 26.54 (CH_2), 30.60 (CH_2), 39.74 (CH_2), 46.39 (CH), 118.56 (CH), 118.68 (CH), 119.65 (C), 121.10 (CH), 124.11 (CH), 130.24 (CH), 131.45 (C), 136.20 (CH), 137.59 (C), 162.93 (C), 210.28 (-C=O) ppm; MS *m/z* = 408 (M^+).

(E)-4-(3,7-dimethylocta-2,6-dienyloxy)-2H-chromen-2-one (***5oc***) [41]: White powder; Yield: 18%, mp 47-48°C; IR (KBr, cm^{-1}): 2923 (Aliphatic C-H), 1718 (C=O), 1620, 1371, 1235 (C-O), 1182 (C-O), 1104 (C-O), 923, 817, 764, 751, 500; ^{1}H-NMR (400MHz,

$CDCl_3$): δ 1.62 (s, 3H, CH_3), 1.69 (s, 3H, CH_3), 1.77 (s, 3H, CH_3), 2.08-2.18 (m, 4H, $-CH_2CH_2-$), 4.71 (d, *J* = 6.7 Hz, 2H, $-CH_2-$), 5.08-5.12 (m, 1H, =CH), 5.49-5.53 (m, 1H, =CH), 5.69 (s, 1H, H-3), 7.25-7.33 (m, H6 and H-8), 7.53-7.57 (m, 1H, H-7), 7.84 (dd, J=2.2Hz, 5.8Hz, 1H, H-5); ^{13}C-NMR (100MHz, $CDCl_3$): δ 16.82 (CH_3), 17.75 (CH_3), 25.70 (CH_3), 26.19 (CH), 39.50 (CH), 66.27 (CH), 90.64 (CH), 115.92 (C), 116.75 (CH), 117.06 (CH), 123.19 (CH), 123.46 (CH), 123.83 (CH), 132.13 (CH), 132.31 (C), 143.74 (C), 153.35 (C), 163.15 (C), 165.61 (C) ppm; MS *m/z* = 298 (M+); Anal. Calcd for $C_{19}H_{22}O_3$: C, 76.48; H, 7.43, Found: C, 76.49; H, 7.40.
